# What Imaging‐Detected Pathologies Are Associated With Shoulder Symptoms and Their Persistence? A Systematic Literature Review

**DOI:** 10.1002/acr.23554

**Published:** 2018-06-06

**Authors:** Gui Tran, Paul Cowling, Toby Smith, Julie Bury, Adam Lucas, Andrew Barr, Sarah R. Kingsbury, Philip G. Conaghan

**Affiliations:** ^1^ University of Leeds Leeds UK; ^2^ Chapel Allerton Hospital Leeds Teaching Hospitals NHS Trust Leeds UK; ^3^ University of Oxford Oxford UK; ^4^ Doncaster and Bassetlaw Teaching Hospitals NHS Foundation Trust Doncaster UK; ^5^ University of Leeds, Leeds, and Arthritis Research UK Centre for Sport, Exercise, and Osteoarthritis Nottingham UK

## Abstract

**Objective:**

Shoulder symptoms are common, and imaging is being increasingly used to help with management. However, the relationship between imaging and symptoms remains unclear. This review aims to understand the relationship between imaging‐detected pathologies, symptoms, and their persistence.

**Methods:**

A systematic review using Medline, EMBASE, Cochrane, and grey literature was conducted to April 2017. The cross‐sectional and longitudinal relationships between imaging‐detected abnormalities and symptoms were analyzed and associations qualitatively characterized by a best‐evidence synthesis based on study design, covariate adjustment, and the Grade of Recommendations Assessment, Development, and Evaluation (GRADE) methodology. Modalities included ultrasound, magnetic resonance imaging (MRI), radiographs, positron emission tomography (PET), bone scintigraphy, and computed tomography.

**Results:**

A total of 6,569 abstracts was screened and 56 articles were included. In total, 50 studies did not adjust for covariates and 36 analyzed individual pathologies only. The majority of studies showed conflicting results. There was no significant association between most imaging features and symptoms among high‐quality, cross‐sectional studies. There was low‐quality evidence that enhancement of the joint capsule on MRI and increased uptake on PET were associated with symptoms in adhesive capsulitis. Based on high‐quality longitudinal studies, enlarging rotator cuff tears were associated with an increased incidence of symptoms.

**Conclusion:**

There were conflicting results on the association of imaging features with shoulder symptoms and their persistence. The existing evidence was very low in quality, based on the GRADE methodology. Further high‐quality studies are required to understand the relationship between imaging and shoulder symptoms and to determine the appropriate role of imaging in care pathways.

## Introduction

Shoulder pain is a very common musculoskeletal condition and a significant contributor to disability and morbidity 1, 2. Recovery can be slow, with high rates of chronic pain; in a community‐based cohort, only 49% of respondents reported a complete recovery at 18 months 3. Shoulder pain has a significant negative impact on quality of life 4, 5, 6. It also poses a significant economic burden, with costs estimated to be £310 million in the first 6 months following primary care contact 7.

**Significance & Innovations**

- The majority of studies show conflicting results on the association of imaging‐detected features with symptoms.
- The majority of studies did not evaluate the role of multiple pathologies in shoulder symptoms.
- Of the possible individual structures to be associated with pain, enlarging rotator cuff tears may be associated with incident symptoms.

Imaging modalities such as ultrasound and magnetic resonance imaging (MRI) can accurately detect soft‐tissue pathologies such as rotator cuff (RC) tears, tendinopathies, and subacromial bursitis 8, 9, and can detect pathology more accurately than clinical examination. To aid the diagnosis of shoulder pain, different imaging modalities have therefore been increasingly used. Despite this increase in imaging, the relationship of imaging findings to patient outcomes remains unclear. A systematic review on the accuracy of imaging has highlighted the fact that further studies are required to determine the extent to which diagnostic tests on shoulder pain ultimately inform patient management and affect outcomes 10. A report by the UK Academy of Medical Sciences has also highlighted the importance of rational, cost‐effective diagnostic tests to improve patient care and reduce costs 11.

The relationship between imaging‐detected shoulder pathologies and clinical symptoms may be complex. Imaging studies have shown that pathologies exist in asymptomatic individuals 12, 13, 14, whereas other studies have suggested that certain features may correlate with pain 15, 16. Our aim was to systematically review the literature to determine what imaging features are associated with symptoms and their progression when common imaging modalities are employed.

## Materials and Methods

### Search strategy and selection process

**Figure 1:**
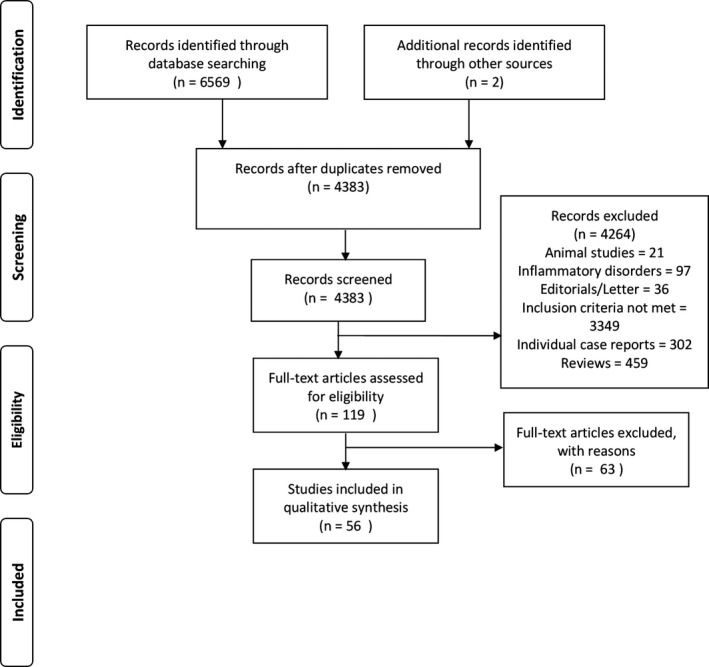
Flow diagram. Reproduced, with permission, from Moher D, Liberati A, Tetzlaff J, Altman DG, for the PRISMA Group. Preferred Reporting Items for Systematic Reviews and Meta‐Analyses: the PRISMA statement. PloS Med 2009;6:e1000097.

The Preferred Reporting Items for Systematic Reviews and Meta‐Analyses methodologies were followed and are described in Figure 1. A systematic literature search of Medline, EMBASE, and the Cochrane library databases until April 2017 was performed. Grey literature and trial registries were searched, including Open Grey, ClinicalTrials.gov, and the World Health Organization International Trials Registry Platform. A full description of the search strategy is shown in Supplementary Table 1, available on the *Arthritis Care & Research* web site at http://onlinelibrary.wiley.com/doi/10.1002/acr.23554/abstract.

Studies were included if they reported the relationship between structural abnormality on imaging and symptoms (cross‐sectional) or progression/persistence of symptoms (longitudinal). Structures included RC tear, tendinopathy, subacromial bursitis, subacromial space, and acromion. Outcome measures included pain or function measures. Imaging modalities included radiographs, ultrasound, computed tomography, MRI, and positron emission tomography (PET). Exclusion criteria were postsurgical patients, systemic inflammatory conditions (such as polymyalgia rheumatica), neurologic disease, chronic pain syndrome, fibromyalgia, and nonhuman studies. There was no language restriction.

### Data extraction

The citations identified by a preliminary search were screened by 2 reviewers (GT and PC) and for references not identified by the preliminary search. Discordance in opinion was resolved by a third reviewer (SRK). Data extraction was performed by 2 reviewers (GT and PC). Articles meeting the inclusion/exclusion criteria were divided into longitudinal and cross‐sectional articles and were evaluated for their relationship to shoulder symptoms and whether single or multiple pathologies were assessed. Extracted data were inclusion criteria and population, patient number/controls, patient demographics (age, sex, and body mass index), study design, aims, imaging feature, symptoms, whether pathology was defined, results with or without adjustment for confounders, and findings.

### Quality assessment

The quality of each observational study was independently assessed by 2 reviewers (GT, PC) (see Supplementary Table 2, available on the *Arthritis Care & Research* web site at http://onlinelibrary.wiley.com/doi/10.1002/acr.23554/abstract). Briefly, a standardized quality scoring tool, previously reported 17, 18, was adapted to assess the following components: study population, imaging feature, pain or function outcome, study design, and analysis and data presentation. A score of 1 or 0 was allocated for each question according to whether the study fulfilled the criteria or not. Any discordance in opinion was recorded, and where consensus could not be achieved, a third reviewer (PGC) was consulted. Quality scores were converted to percentages of the maximum scores for each class of article (cross‐sectional, case–control, or cohort study). A study was considered to be high quality if it exceeded or equaled the mean score in its class.

Meta‐analysis was inappropriate due to heterogeneity in study populations and imaging modalities. A narrative analysis of the evidence for features and their associations with symptoms was provided, based on the study design adequacy of adjustment for covariates, using a best‐evidence synthesis approach 19. Comparisons were made for cross‐sectional studies and longitudinal studies. The research synthesis results were interpreted using the Grade of Recommendations Assessment, Development and Evaluation (GRADE) framework 20.

## Results

### Systematic literature search and selection

Following exclusion of duplicates, 4,383 articles were included, of which 119 met the inclusion/exclusion criteria and were screened. In total, 56 articles were included (41 cross‐sectional, 11 cohort, 4 case–control). Imaging modalities included 25 ultrasound 16, 21, 22, 23, 24, 25, 26, 27, 28, 29, 30, 31, 32, 33, 34, 35, 36, 37, 38, 39, 40, 41, 42, 43, 44, 24 MRI 15, 16, 41, 44, 45, 46, 47, 48, 49, 50, 51, 52, 53, 54, 55, 56, 57, 58, 59, 60, 61, 62, 63, 64, 12 radiographs 27, 30, 49, 53, 55, 62, 65, 66, 67, 68, 69, 70, 3 bone scintigraphy 65, 71, 72, 2 PET 73, 74, and no computed tomography. Of these studies, 10 assessed associations with 2 imaging modalities (16, 27, 30, 41, 44, 49, 53, 55, 62, 65). Most studies included both sexes; 8 studies did not state the sex ratio involved 35, 38, 41, 59, 62, 65, 71, 74, and 1 included males only 34. The nomenclature for defining imaging pathologies varied between studies, and there was no standardized way of defining pathology (see Supplementary Table 3, available on the *Arthritis Care & Research* web site at http://onlinelibrary.wiley.com/doi/10.1002/acr.23554/abstract). There was heterogeneity between study populations.

### Summary of the methodologic qualities of the studies included

Supplementary Table 2, available on the *Arthritis Care & Research* web site at http://onlinelibrary.wiley.com/doi/10.1002/acr.23554/abstract, shows the results of the methodologic quality assessment of the included studies. The mean (range) score was 48% 28, 29, 30, 31, 32, 33, 34, 35, 36, 37, 38, 39, 40, 41, 42, 43, 44, 45, 46, 47, 48, 49, 50, 51, 52, 53, 54, 55, 56, 57, 58, 59, 60, 61, 62, 63, 64, 65, 66, 67 for cohort, 41% 7, 8, 9, 10, 11, 12, 13, 14, 15, 16, 17, 18, 19, 20, 21, 22, 23, 24, 25, 26, 27, 28, 29, 30, 31, 32, 33, 34, 35, 36, 37, 38, 39, 40, 41, 42, 43, 44, 45, 46, 47, 48, 49, 50, 51, 52, 53, 54, 55, 56, 57, 58, 59, 60, 61, 62, 63, 64, 65, 66, 67, 68, 69, 70, 71 for cross‐sectional, and 50% 47, 48, 49, 50, 51, 52, 53, 54, 55, 56, 57, 58, 59 for case–control studies. A total of 31 studies were high quality, and 25 were low quality. Six studies did not explain the statistical test used 33, 42, 46, 52, 58, 71. Nine studies did not define pathology. The GRADE quality of evidence for the relationship for all imaging features/symptoms outcomes was very low because of study limitations (risk of bias and observational study design), quality, inconsistency, and indirectness.

### Cross‐sectional relationship between individual pathology features and symptoms

#### RC tears

Sixteen studies evaluated the relationship of RC tears and symptoms (16, 21, 26, 29, 30, 32, 35, 45, 48, 49, 52, 54, 56, 58, 62, 72) (Tables 1, 2, and 3). Nine evaluated the relationship with shoulder pain 16, 21, 30, 32, 35, 45, 49, 52, 72, 4 with shoulder disability 16, 29, 45, 54, and 8 studies with symptoms using a composite pain and function score 26, 29, 32, 48, 49, 54, 56, 58.

**Table 1:** Cross‐sectional ultrasound scans^a^

Author, year (ref.)	Patient and study characteristics	Findings	Quality score
Ardic, 2006 16, b	Clinically suspected SIS; secondary care; 58 patients/no controls; 13 males; mean age 55.5 years	SAB effusion/hypertrophy correlated with shoulder extension pain (r = −0.04, *P* = 0.03); complete supraspinatus tear correlated with pain on internal (r = 0.4, *P* = 0.04) and external rotation (r = 0.3, *P* = 0.02); subacromial bursa effusion/thickening was correlated with restricted shoulder internal rotation (r = −0.4, *P* = 0.02); after applying logistic regression it was found that only glenoid labral tear and bursal effusion/hypertrophy on MRI were determinants of shoulder disability	43
Brasseur, 2004 21, b	Tennis players from French veteran championship of the Roland Garros Tennis Open; 150 consecutive patients/contralateral shoulder; 85 men; mean age 55 years	SAB effusion or thickness >2 mm associated with pain (*P* < 0.001); a complete supraspinatus tendon tear occurred significantly more frequently in players with current pain and those with former pain (*P* < 0.05); no relationship between calcification and pain	36
Chiou, 2002 22, c	Shoulder calcification on radiographs; population NR; 94 patients; 42 male; average age 57 years	Significant difference between the morphology of the calcific plaques and the clinical symptoms (*P* < 0.01) (non–arc‐shaped calcifications had more severe symptoms); high‐grade color Doppler had significantly increased severe symptoms (*P* < 0.01)	29
Cholewinski, 2008 23, b	Clinical SIS; orthopedic outpatients; 57 patients /unaffected contralateral shoulder/36 asymptomatic volunteers; 23 males; mean age 56 years	Difference in distance (3.3 mm) between acromion and the AGT of humerus in affected joints and controls, and 2.1 mm in comparison to the contralateral unaffected joint (*P* = 0.000001); significantly reduced AGT distance in affected joints (*P* < 0.000001); RC thickness not statistically significant between affected and control but was significant between affected and unaffected shoulder of the same patient (1.1 mm) (*P* < 0.000001)	43
Daghir, 2012 24, b	Clinical SIS; recruited from university hospital; 22 patients/23 healthy; 10 male; mean age 52 years	Bursal fluid thickness significantly greater in SIS when measured using the short‐axis supraspinatus view only (*P* < 0.006); peribursal fat was significantly thicker in all patients than controls on the long‐axis subscapularis view only (*P* = 0.036); SAB dynamic bunching not associated with SIS symptoms (*P* = 0.41)	29
Draghi, 2015 25, c	US of shoulders; radiology department 1,105 consecutive patients/none; 600 males; mean age 52 years	Effusion in the SASD bursa was associated with shoulder pain independent from the underlying pathology (*P* < 0.01)	36
Fehringer, 2008 26	Patient ages >65 years from orthopedic lower extremity clinic; 104 patients/number not specified (those without RC tears and not seen by a physician); 53 males; mean age 71.4 years	Mean Constant scores were lower for those with full‐thickness tears than for those without after adjusting for age and sex (*P* = 0.0003); for those without tears, odds of having an SST score of 9 or greater were 0.22 times those with tears (*P* < 0.0001)	43
Hamid, 2012 27, c	Asymptomatic RC tears; population NR; 216 patients/47 (no RC tear) (43 people with no RC tear were used as a control for AI); 128 males; average age 64.8 years	Acromion index associated with pain (*P* = 0.02); no significant difference between acromion spur and pain; presence of an acromial spur, regardless of size, was highly associated with a full‐thickness RC tear, even after adjusting for age, sex, and hand dominance (OR 3.05 [95% CI 1.42–6.52])	50
Joensen, 2009 28, b	Clinical diagnosis of tendinopathy; general practice and physiotherapy outpatients; 64 patients/64 asymptomatic contralateral side; 28 males; mean age 47.5 years	For symptomatic side, maximal pain‐free isometric force (≤10 N), tendon pain pressure (≥0.6 kg), and tendon thickness (≥0.8 mm) significantly different compared to asymptomatic side (*P* < 0.001)	50
Keener, 2009 30, b	Unilateral shoulder pain related to RC disease; 62 (symptomatic side)/98 (asymptomatic side); background population not stated; 32 males; mean age 60.6 years	Humeral migration was related to tear size in symptomatic patients with a critical size of tear >175 mm^2^ related to humeral migration (*P* = 0.01); proximal humeral migration was greater in the shoulders with a symptomatic tear (*P* = 0.03); no difference in VAS between small and large RC tears in the symptomatic group; no significant difference in RC tear size between the asymptomatic and symptomatic shoulders; in the symptomatic group with full RC tears >175 mm^2^, VAS was correlated with tear size (r = 0.70, *P* = 0.001) and humeral migration (r = 0.68, *P* = 0.002)	43
Keener, 2010 29, c	Symptomatic RC tear and contralateral asymptomatic RC tear; orthopedic department patients; 196 patients/54 (intact RC); 118 males; mean age 62.1 years	RC tear (partial or full) associated with a clinically insignificant loss of shoulder function; no differences were seen in functional scores between different sizes of full‐thickness RC tears	43
Le Goff, 2010 31, c	Calcific tendonitis on radiograph; rheumatology outpatient; 57 consecutive patients/24 (asymptomatic calcific tendonitis); 19 men; mean age 51 years	Power Doppler within the calcific deposit and widened SAB (>2 mm) associated with pain (*P* < 0.005); larger (*P* = 0.0015) and fragmented (*P* = 0.01) calcifications were associated with pain	50
McMahon, 2014 32, b	Elite athletes participating in 2005 Senior Olympics; ages >60 years; 141 patients/no controls; 58 men; median age 70	Increased odds of pain VAS score with RC abnormality (tear or tendinopathy) (OR 8.0 [95% CI 1.0–62.5]); pain not associated with types of pathology (full or partial RC tear); ASES and DASH not related to US findings	36
Tracy, 2012 33, b	Clinical suspicion of coracoid impingement; population NR; 7 patients/19 (asymptomatic); 6 males; mean age 55.9 years	CHI is significantly narrower in symptomatic shoulders than in asymptomatic volunteers (*P* < 0.0001)	43
Wu, 2010 34, b	Clinical suspected SIS; high school players; 10 patients/16 (asymptomatic); 10 males; mean age 16.7 years	Significant displacement found in CAL in symptomatic patients (mean 3.0 mm) (*P* = 0.017)	57
Yamaguchi, 2006 35, b	Unilateral shoulder pain; population NR; 58 patients/no controls; sex distribution not stated; average age 62.8 years	In patients with bilateral RC tears, increased size may be associated with pain (*P* < 0.01) (mean size for asymptomatic = 17.4 mm vs. 22.7 mm symptomatic)	36

a SIS = subacromial impingement syndrome; SAB = subacromial bursa; MRI = magnetic resonance imaging; NR = not reported; AGT = apex of the greater tuberosity; RC = rotator cuff; US = ultrasound; SASD = subacromial subdeltoid; SST = Simple Shoulder Test; AI= acromion index; OR = odds ratio; 95% CI = 95% confidence interval; VAS = visual analog score; ASES = American Shoulder and Elbow; DASH = Disabilities of the Arm, Shoulder and Hand score; CHI = coracohumeral interval; CAL = coracoacromial ligament.

b Studied symptoms as independent variables and imaging features as dependent variable.

c Studied imaging features as independent variables and symptoms as dependent variable.

**Table 2:** Cross‐sectional MRI scans^a^

Author, year (ref.)	Patient and study characteristics	Findings	Quality score
Ahn, 2012 15, b	Clinical adhesive capsulitis; orthopedic surgery department patients; 97 patients/no controls; 47 males; mean age 56 years	Thickening of joint capsule in axillary recess associated with decreased ER in males, in their nondominant arm (r^2^ = 0.34, *P* < 0.05); gadolinium enhancement of the joint capsule in axillary recess correlated with pain intensity (OR 0.78 [95% CI 0.62–0.97]; *P* < 0.05); no significant correlation between subcoracoid fat obliteration of the rotator interval or supraspinatus pathology and shoulder ROM or pain	50
Ardic, 2006 16, b	Clinically suspected SIS; secondary care patients; 59 shoulders; 13 males; mean age 55.5 years	Severity of disability correlated with SAB effusion (r = 0.4, *P* = 0.03) and labral tear (r = 0.5, *P* = 0.02); labral tears associated with pain (r = 0.8, *P* = 0.00) and disability (r = 0.6, *P* = 0.02); SAB effusion associated with disability (r = 0.5, *P* = 0.03); restricted movements associated with RC tears	43
Birtan, 2001 64, b	SIS (defined by improvement to local anesthetic injection); 86 patients; 48 males; average age 51.6 years	Stage 3 tendinopathy significantly associated with worse score (*P* < 0.05)	36
Curry, 2015 45, c	RC tears; orthopedic and physiotherapy clinics; 67 patients/no controls; 37 males; 58% ages >60 years	Pain and function status were not associated with tear size/thickness, fatty infiltration, and muscle atrophy	71
Di Mario, 2005 46, b	Clinical SIS; background population not stated; 74 patients with SIS/no controls; 47 males; mean age 49 years	Impingement syndrome is positively correlated to intrinsic acromial angle and negatively correlated to acromiohumeral distance	36
Epstein, 1993 47, b	Surgically proven SIS; surgically proven RC tears; background NR; 30 SIS (6 men, mean age 39 years)/35 cuff tears (25 men, mean age 58 years)/56 controls (26 males, mean age 36 years)	Patients with RC tears had increased prevalence of type 3 acromion compared to control, and in impingement group (*P* < 0.001)	36
Frost, 1999 48, b	Clinical SIS; population NR; 42 patients/31 controls; 25 males; mean age 47.5 years	No association between supraspinatus pathology and pain	59
Gill, 2014 49, b	Current shoulder pain, history of shoulder pain, and no history of shoulder pain; general population in Australia; 30 in total: 10 current shoulder pain, 10 history of shoulder pain, and 10 no history of shoulder pain; 12 males; mean age 64.8 years	No significant differences in shoulder pathologies and those with/without pain	43
Graichen, 1999 50, b	Clinical SIS; population NR; 10 patients/10 controls; 5 males, ages 39–64 years	A significant decrease in the width of the subacromial space compared with that of the healthy contralateral side during activity (*P* < 0.05)	47
Hodgson, 2012 51, c	RC tears; primary care referrals to the shoulder ultrasound service; 18 with pain/15 without pain; 5 males; mean age 55.4 years	No link between pain and bursal enhancement (OR 20.44 [95% CI 0.03–22,347.73]; *P* = 1.00)	57
Jung, 2013 52, c	Arthroscopic confirmed full‐thickness tear of the subscapularis tendon; orthopedic referrals; 29 patients/no controls; 11 males; mean age 64.5 years	Patients with the bridging sign had longer duration of shoulder pain (no statistical significance given)	7
Kanatli, 2011 53, c	Clinical SIS; orthopedic department; 44 patients/no controls; patients scheduled for shoulder arthroscopy; 20 males; mean age 54.1 years	No correlation between radiologic measurements and severity of acromial impingement	71
Krief, 2004 54, b	Mainly pain in deltoid region after the failure of noninflammatory therapy and a rehabilitation program; patients referred by sports medicine clinicians or orthopedic surgeons; 1,075 patients/no controls; 47% male; mean age 52 years	The presence, size, and location of full‐thickness RC tears did not influence the level of disability or pain; the global disability was statistically linked to partial thickness tears involving the superficial and deep surfaces of the supraspinatus tendon (*P* < 0.01, R^2^ = 0.350), to the presence of bursitis (*P* = 0.01 R^2^ = 0.337), but not other RC or biceps pathology	43
Mayerhoefer, 2009 55, b	Clinical SIS failed to respond to treatment for >6 months; orthopedic department 47 patients/no controls; 33 males; mean age 51.7 years	The Constant score was correlated with AHD (r = 0.39 for radiograph and 0.41 for MRI, *P* < 0.01) but not with acromial shape; patients with an AHD <7 mm on MRI had significantly lower Constant scores than those with an AHD >7 mm (mean difference 18.5; *P* < 0.01)	57
Moses, 2006 57, b	Patients with surgically diagnosed impingement and instability; secondary care; 27 GH instability, no impingement; 18 shoulder impingement, no tear; 21 impingement with tear/no controls; 48 males; mean age 29 years	No difference in scapula position between instability, impingement with tears or impingement without tears	42
Reuter, 2008 58, b	Symptomatic or nonsymptomatic athletes; Ironman triathletes; 16 patients/7 (asymptomatic)/17 nonathletes; 11 males; average age 39 years	No statistical difference in prevalence in RC tendinopathy/tears or ACJ disease	36
Schweitzer, 1995 59, c	Criteria and background population not defined; 208 patients with mixture of shoulder pathology/17 controls; sex ratio NR; mean age 47 years	GH fluid not associated with focal tenderness, joint pain, or impingement	47
Song, 2011 60, b	Clinically diagnosed adhesive capsulitis; patients attending radiology department; 35 patients/45 controls; 14 males; mean age 50.1 years	Thicker joint capsule in the axillary recess and thicker enhancing portion of the axillary recess and the RC interval associated with adhesive capsulitis (*P* < 0.001)	50
Unruh, 2014 56, b	Symptomatic RC tears; enrolled by surgeons involved 450/no controls; full‐thickness cuff tears; 49% male; mean age 62 years	Longer duration of symptoms does not correlate with more severe cuff disease; duration was unrelated to weakness, decreased ROM, tear size, fatty atrophy, muscle retraction AHD, or validated outcome measures	43
White, 2006 61, c	Patients with full thickness RC tears; population NR; 35 patients/36 asymptomatic; 22 males; mean age 41 years	Mean SAB thickness in symptomatic individuals significantly higher than in asymptomatic in RC tears (3.3 mm vs. 1.3 mm, respectively; *P* < 0.05) and fluid in symptomatic patients located in the anterior quarter of the humerus or anterior to the humerus	21
Williamson, 1994 62, b	Clinical diagnosis of impingement syndrome, based on relief of symptoms after lidocaine injections; population NR; 41 participants with impingement syndrome/40 patients with shoulder instability used as controls; sex ratio NR; mean age 39 years	Absence of subacromial fat, presence of a supraspinatus tear, subacromial osteophytes, and a decreased coracohumeral distance observed in impingement compared to shoulder instability groups	14

a MRI = magnetic resonance imaging; ER = external rotation; OR = odds ratio; 95% CI = 95% confidence interval; ROM = range of motion; SIS = subacromial impingement syndrome; SAB = subacromial bursa; RC = rotator cuff; NR = not reported; AHD = acromiohumeral distance; GH = glenohumeral; ACJ = acromion clavicular joint.

b Studied symptoms as independent variables and imaging features as dependent variable.

c Studied imaging features as independent variables and symptoms as dependent variable.

**Table 3:** Cross‐sectional radiograph, PET, and bone scans^a^

Author, year (ref.), test	Patient and study characteristics	Findings	Quality score
Binder, 1984 65, radiograph^b^	Clinical adhesive capsulitis; population NR; 42 had radiographs/40 controls; patients with capsulitis; age and sex not documented	No association was found between the passive range or its recovery and the findings on plain radiograph	21
Endo, 2001 66, radiograph^c^	Clinically diagnosed chronic SIS; orthopedic outpatient clinic; 27 patients/7 controls; 14 males; mean age 57.5 years	Upward and axial rotational tilts of scapula impaired in shoulder pain (*P* < 0.05)	47
Gill T, 2014 49, radiograph^c^	Current shoulder pain, history of shoulder pain, and no history of shoulder pain; general population in Australia; 30 in total: 10 current shoulder pain, 10 history of shoulder pain, and 10 no history of shoulder pain; 12 males; mean age 64.8 years	No significant differences in shoulder pathologies and those with/without pain	43
Hamid, 2012 27, radiograph^b^	Asymptomatic RC tears; orthopedic department; 216 patients/47 (contralateral asymptomatic RC intact side); 128 males; mean age 68.4 years	Acromion index associated with the development of pain (*P* = 0.02); no significant difference between acromion spur and development of pain; presence of an acromial spur, regardless of size, was highly associated with a full‐thickness RC tear, even after adjusting for age, sex, and hand dominance (OR 3.05 [95% CI 1.42–6.52])	50
Kanatli, 2011 53, radiograph^b^	Clinical SIS; orthopedic department; 44 patients/no controls; 20 males; mean age 54.1 years	No correlation between radiologic measurements and severity of acromial impingement	71
Keener, 2009 30, radiograph^c^	Unilateral shoulder pain related to RC disease; background population NR; 62 (symptomatic side)/98 (asymptomatic side); 32 males; mean age 60.6 years	Humeral migration is related to tear size in symptomatic patients with a critical size of tear >175 mm^2^ related to humeral migration (*P* = 0.01); no difference in VAS between small and large RC tears in the symptomatic group; in groups with ≥175 mm^2^ tear, there was a correlation between VAS and migration (r = 0.68, *P* = 0.002) and VAS and the tear area (r = 0.70, *P* = 0.001) pain and tear area predictors of humeral migration (overall model r^2^ = 0.63, *P* = 0.0006), with the tear area to be the single most important (r^2^ = 0.63, *P* = 0.01)	43
Kircher, 2010 67, radiograph^b^	Advanced OA of the shoulder; background population NR; 120 patients/no controls; 64 males; mean age 64.9 years	Increasing size of osteophytes is correlated to reduced active and passive range of motion: flexion (r = −0.203, *P* = 0.026; r = −0.254, *P* = 0.026, respectively), abduction (r = −0.197, *P* = 0.032; r = −0.270, *P* = 0.017), external rotation (r = −0.243, *P* = 0.008; r = −0.338, *P* = 0.002), and internal rotation (r = −0.243, *P* = 0.008; r = −0.245, *P* = 0.030); joint space width not associated with pain or ROM	43
Kircher, 2012 68, radiograph^b^	Calcific tendinitis on radiographs; orthopedic department; 109 patients/no controls; 46 males; mean age 48.2 years	No association or correlation between acromion index/calcium deposition and pain or function	50
Mayerhoefer, 2009 55, radiograph^c^	SIS; orthopedic department; 47 patients/no controls; 33 males; mean age 51.7 years	The Constant score was correlated with AHD (r = 0.39 for radiograph and 0.41 for MRI, *P* < 0.01) but not with acromial shape; patients with an AHD <7 mm on MRI had significantly lower Constant scores than those with an AHD >7 mm (mean difference, 18.5; *P* < 0.01)	57
Williamson, 1994 62, radiograph^c^	Clinical diagnosis of impingement syndrome, based on relief of symptoms after lidocaine injections; background population NR; 41 participants with impingement syndrome/40 patients with shoulder instability used as controls; sex ratio NR; mean age 39 years	Subacromial osteophytes, but not sclerosis and cysts, observed in SIS group vs. control	14
Yamaguchi, 2000 69, b	Full‐thickness RC tears; population NR; 10 painful shoulders/10 asymptomatic tears/10 normal volunteers; 5 males with painful shoulders; age range 20–29 years (mean age not given)	Although RC tears demonstrated abnormal GH kinematics, there was no relationship with symptoms	36
Kim, 2013 73, PET scan^c^	Patients diagnosed with adhesive capsulitis in musculoskeletal pain clinic; 22 shoulders in 21 patients, 40 shoulders in 20 patients (control group); 9 males; mean age 59.3 years	Specific patterns of uptake in the rotator interval, ACJ, or axillary recess may be related to adhesive capsulitis; increased uptake of ^18^F‐FDG in RI, AJC, or AR compared to controls and contralateral shoulder (*P* < 0.001)	43
Sridharan, 2017 74, b	Adhesive capsulitis; population not documented; 15 patients with confirmed adhesive capsulitis/109 controls; patients with capsulitis; age and sex not documented	Significant association with PET positivity, and AC was significant (Fisher's exact test, *P* = 0.001).	14
Binder, 1984 65, bone scan^b^	Clinical adhesive capsulitis; population not documented; 38 had bone scans/40 (similar age/sex no symptoms); patients with capsulitis; age and sex not documented	No association between technetium uptake and duration of symptoms, initial severity, or recovery; significantly increased technetium uptake in symptomatic shoulder compared to contralateral asymptomatic shoulder or controls (*P* < 0.0001)	21
Clunie, 1998 71, bone scan^c^	Unilateral shoulder pain: either clinically diagnosed SIS or adhesive capsulitis; recruited from rheumatology clinic; 12 subacromial impingement; 4 adhesive capsulitis/16 controls (contralateral asymptomatic side); age and sex NR	No difference in Tc‐HIG distribution between symptomatic vs. asymptomatic shoulders	14
Koike, 2013 72, bone scan^c^	Symptomatic cuff tears; secondary care hospital; 28 symptomatic tear, 26 asymptomatic cuff tear/20 no tear (controls); 14 males; mean age 62 years	Shoulders with a symptomatic RC tear showed higher radioisotope uptake on bone scintigraphy than those with an asymptomatic tear, or shoulders without tears (*P* = 0.02)	50

a PET = positron emission tomography; NR = not reported; SIS = subacromial impingement syndrome; RC = rotator cuff; OR = odds ratio; 95% CI = 95% confidence interval; VAS = visual analog score; OA = osteoarthritis; ROM = range of motion; AHD = acromiohumeral distance; MRI = magnetic resonance imaging; GH = glenohumeral; ACJ = acromion clavicular joint; RI = rotator interval; AR = axillary recess; AC = adhesive capsulitis; Tc‐HIG = Technetium‐99m human immunoglobulin imaging.

b Studied imaging features as independent variables and symptoms as dependent variable.

c Studied symptoms as independent variables and imaging features as dependent variable.

RC tear was associated with pain in 4 ultrasound studies 16, 21, 32, 35, 3 MRI studies 16, 52, 62, and 1 bone scintigraphy study 72. These studies were unadjusted, and the majority were of low quality. Larger RC tear size (mean size 22.7 mm) was associated with pain in 1 low‐quality study 35. One study reported no association of RC tear size and symptoms, although in symptomatic tears >175 mm^2^, pain was correlated with tear size 30. Two high‐quality studies reported no association between RC tear size or location with pain, one of which was well‐adjusted 45, 48. The type of RC tear (partial or full) was not associated with severity of pain 32.

RC tear was associated with disability in 2 high‐quality MRI studies 16, 54. RC tear was not associated with functional disability in 1 high‐quality ultrasound study 16 and 2 high‐quality MRI studies, one of which was well‐adjusted 45, 49. One study reported an association of RC tears with disability on MRI but not on ultrasound 16. RC tear was associated with worse composite scores in 2 ultrasound studies 26, 32. There was no association between RC tears and composite scores in 2 ultrasound studies 29, 32 and 5 MRI studies 48, 49, 54, 56, 58. In summary, in 1 high‐quality, well‐adjusted study, RC tears were not associated with pain or function 45. The other studies were of mixed quality, unadjusted, and reported conflicting findings.

#### Tendinopathies

Eight studies evaluated tendinopathy and symptoms 16, 23, 28, 32, 48, 49, 58, 64 (Tables 1, 2, and 3). Two evaluated the relationship with pain 32, 48, 3 with disability 16, 28, 49, and 7 with both pain and disability 23, 28, 32, 48, 49, 58, 64. One high‐quality ultrasound study reported an association using measures of both pain and composite symptoms, although the authors did not separate those with tendinopathies from those with RC tears 32. In 1 high‐quality study, tendinopathy on MRI was not related to clinical shoulder impingement (pain only) 48. One high‐quality ultrasound study reported a relationship between tendinopathies and disability 28. One high‐quality ultrasound study 16 and 1 high‐quality MRI study 49 reported no relationship.

One ultrasound study reported a relationship between RC tendon thickness (≥0.8 mm) and symptoms, which were undefined 28. One low‐quality MRI study found that only a high‐stage tendinopathy, defined by complete disruption of supraspinatus tendon, was associated with symptoms 64. There was no association between RC thickness and symptoms of impingement (pain with functional impairment) between patients in 1 ultrasound study, but a significant difference in RC thickness of >1.1 mm was seen between affected and unaffected shoulders of the same patient 23. Three MRI studies of mixed quality 48, 49, 58 reported no relationship with tendinopathy and symptoms. In summary, high‐quality but unadjusted studies found a conflicting relationship between pain, disability, and tendinopathy 16, 28, 32, 48, 49. No studies adjusted for covariates.

#### Subacromial bursal pathology

Ten studies (16, 21, 24, 25, 31, 49, 51, 53, 54, 61) evaluated the relationship between the subacromial bursa (SAB) and symptoms (Tables 1, 2, and 3). Five mixed‐quality ultrasound studies 16, 21, 24, 25, 31 and 2 MRI studies of mixed quality 16, 61 reported an association between SAB and pain. In 1 study, peribursal fat and fluid and bursal thickness, but not bunching, were associated with pain 24. One study reported an association with pain and SAB when seen alongside power Doppler within calcific deposits 31. One study reported that the location of bursa pathology was important 61. One ultrasound study 16 and 1 MRI study 16 reported that SAB effusion/thickening was associated with reduced function. Two high‐quality MRI studies reported no association 49, 53. One high‐quality MRI study reported an association between bursitis and symptoms 54. Two high‐quality MRI studies reported no relationship between SAB enhancement and composite score 49, 51. In summary, 2 high‐quality, unadjusted studies found no relationship between shoulder symptoms and subacromial pathology 51, 53. No studies adjusted for covariates.

#### Osteoarthritis (OA)

Four studies of mixed quality 49, 58, 62, 67 evaluated the relationship between shoulder OA and symptoms (Tables 1, 2, and 3). One low‐quality combined MRI and radiographic study reported an association between subacromial osteophytes in patients with impingement 62. One high‐quality radiographic study reported no relationship between acromioclavicular joint (ACJ) or glenohumeral joint (GHJ) OA and pain 49, and another no relationship with GHJ space width 67. One radiographic study reported that an increased size of osteophytes, but not joint space, was correlated with reduced range of motion 67. Two MRI studies of mixed quality reported no relationship between pain, function, and ACJ arthrosis 49, 58. In summary, 1 high‐quality, unadjusted study found no relationship with symptoms and features of ACJ or glenohumeral OA 49. There were no adjusted studies.

#### Calcification

Three studies evaluated the association between calcification and pain 21, 22, 31, and 2 studies evaluated the association with pain and function 49, 68 (Tables 1, 2, and 3). Two ultrasound studies of mixed quality 22, 31 showed that calcification was associated with pain, and 1 low‐quality study found no association 21. Larger fragmented calcifications (mean dimensions: longitudinal 1.64 cm and transverse 1.39 cm) were associated with pain 31, as was morphology and color Doppler 22. Two high‐quality radiographic studies reported no association with pain or function 49, 68. In summary, 2 high‐quality, unadjusted studies found no relationship with calcification and symptoms 49, 68. There were no adjusted studies.

#### Acromion pathology

Twelve studies (23, 27, 34, 46, 47, 50, 53, 55, 56, 57, 62, 68) evaluated the relationship between the acromion and symptoms (Tables 1, 2, and 3). Two ultrasound studies 23, 34, 3 MRI studies 47, 50, 57, 1 radiographic study 68, 2 combined MRI and radiographic studies 53, 62 and 1 combined ultrasound and radiographic study 27 evaluated the relationship of the acromion and pain. One study radiographically evaluated the relationship with function 68. Two MRI studies 46, 56 and 1 combined MRI and radiographic study 55 evaluated the relationship with symptoms using a composite score.

One high‐quality combined radiographic and ultrasound study 27 reported that the acromial index (lateral extension of the acromion relative to humeral head) was associated with pain, whereas 2 high‐quality radiographic studies 53, 68 reported no association with pain or function. Those with full‐thickness cuff tears had an increased prevalence of type 3 acromion compared to controls and patients with surgical impingement 47. No relationship existed between scapuloacromial angle 57, subacromial distance, or acromion shape 62 and impingement. One MRI study in patients with clinical impingement reported a reduction in the subacromial space during activity 50, and another reported decreased coracohumeral distance 62.

One high‐quality, adjusted ultrasound study reported displacement in the coracoacromial ligament in symptomatic patients 34. A difference in distance (2.1 mm) between the inferolateral edge of the acromion and the apex of the greater tuberosity of the humerus was observed in affected shoulders in a high‐quality study 23. Two studies of mixed quality reported an association with acromial humeral distance (AHD) and symptoms 46, 55, but another high‐quality study found no association 56. One study showed no relationship with acromial shape 55, whereas another was positively correlated to the intrinsic acromial angle 46.

In summary, high‐quality, unadjusted studies found conflicting results on the relationship between symptoms and the acromial index (lateral extension of the acromion relative to humeral head) 27, 53, 68 and AHD 55, 56. No relationship was found between scapuloacromial angle or acromion shape in high‐quality, unadjusted studies 55, 57.

#### Adhesive capsulitis

Six studies 15, 60, 65, 71, 73, 74 evaluated the relationship between adhesive capsulitis and pain (Tables 1, 2, and 3). Two high‐quality MRI studies reported enhancement of the joint capsule in the axillary recess, and RC interval was associated with pain intensity 15, 60. One MRI study showed that capsular thickening was associated with decreased external rotation 15. Two PET studies of mixed quality showed increased uptake of ^18^F‐labeled fluorodeoxyglucose (^18^F‐FDG) in the RC interval, anterior joint capsule, or axillary recess 73, 74. One low‐quality bone scintigraphy study reported no difference 71. One low‐quality study using bone scintigraphy and radiographs showed increased technetium uptake but no association between passive range of motion or recovery 65. In summary, high‐quality, unadjusted studies have shown imaging features associated with symptoms in adhesive capsulitis 15, 60, 73.

#### Other features

Several studies evaluated other pathology imaging features. None of these were adjusted. Radiographically, 1 high‐quality study showed that reduced upward and axial rotational tilts of the scapula were impaired in shoulder pain 66. There was no relationship in abnormal scapular planar glenohumeral motion measured using radiographs in patients with RC tears and pain in a low‐quality study 69. One high‐quality MRI study reported that the presence of glenohumeral effusion was not related to pain 59. One high‐quality radiographic study found that in symptomatic patients with full RC tears >175 mm^2^, pain was correlated with humeral migration 30. One low‐quality study reported an association between the absence of subacromial fat in patients with impingement 62. One high‐quality study reported no association between acromioglenoid angle, supraspinatus‐glenoid angle, and pain 53. Glenoid‐labral tear was associated with disability on MRI in a high‐quality study 16. In another high‐quality MRI study, glenoid‐labral tears or cartilage damage were not associated with pain or functional impairment 49. In 1 high‐quality ultrasound study, coracohumeral interval was significantly narrower in symptomatic shoulders (*P* < 0.0001) 33.

### Longitudinal relationship between individual shoulder features and symptoms

#### RC tears

Six ultrasound studies evaluated the relationship between RC tears and symptoms 39, 40, 41, 42, 43, 44 (Tables 4 and 5). Five studies evaluated the relationship between RC tears and pain persistence or progression 39, 40, 42, 43, 44, 3 with function progression 39, 40, 44, and 4 with symptom progression using composite scores 39, 41, 42, 44.

**Table 4:** Longitudinal ultrasound scans^a^

Author, year (ref.)	Patient and study characteristics	Findings	Quality score
Chiou, 2001 36, b	Radiographic calcific tendinosis; recruitment population NR; 100 patients/no controls; 52 males; average age 60 years	Higher vascularity significantly associated with spontaneous resorption and improvement of symptoms (*P* < 0.001); those with arc‐shaped calcific plaque were less likely to resolve spontaneously	39
Couanis, 2015 37, b	Swimmers intending to complete an unassisted channel crossing and between 18–65 years; 22 patients/no controls; 15 males; mean age 37.27 years	SAB thickness is significantly (*P* = 0.05) correlated (β = 0.11) with kilometers swum in the pool in the preceding week; SAB thickness associated with pain 1 week post‐swim (*P* = 0.032), but not prior to race; significant differences in pain between those with severe and normal (*P* = 0.004), mild (*P* = 0.008), or moderate (*P* = 0.012) RC tendinopathy	56
Desmeules, 2004 38, c	Clinically diagnosed SIS; primary care and physical therapy units; 7 patients/13 controls; sex ratio NR; average age 44 years	No difference between AHD and WORC (*P* = 0.06); reduction of AHD narrowing on abduction correlated with improvement of WORC post‐rehabilitation (r = 0.86; *P* = 0.01)	61
Keener, 2015 39, b	Symptomatic RC tear and contralateral asymptomatic RC tear; orthopedic department; 224 patients/36 controls (no RC tears); 112 males; mean age 62 years	RC tear enlargement (>5 mm or change in tear type) associated with a greater risk of pain development (*P* < 0.05); baseline SST (*P* < 0.05) and ASES (*P* < 0.05) scores worsened with advancing tear type; shoulders with new pain had a significant decline in function from baseline (*P* < 0.05); greater risk for pain development associated with advanced final tear type (partial or full thickness) (*P* < 0.05)	56
Mall, 2010 40, b	Asymptomatic RC tear; population NR; 44 patients/55 controls (asymptomatic RC tears); 30 males; mean age 63.3 years	The size of a full‐thickness RC tear increased significantly in those who developed pain (median area increase of 31 mm^2^; *P* = 0.006); larger RC tears on enrollment were more likely to develop pain; function decreased with onset of pain; pain development in asymptomatic RC tears is not associated with progression of fatty degeneration of the RC muscles; no significant differences were seen in GH kinematics and the onset of pain	61
Moosmayer, 2013 41, b	Asymptomatic tears or patients with contralateral shoulder pain; orthopedic outpatients; 50 asymptomatic shoulders; age and sex ratio NR	Tear size increase not associated with the development of symptoms (*P* > 0.05); increased odds of development of symptoms with new biceps tendon pathology (OR 7.5 [95% CI 1.3–42.5]; *P* = 0.02)	67
Moosmayer, 2017 44, b	Patients reviewed by a single orthopedic surgeon in a Norwegian secondary care center; 49 patients; 30 males; average age 61 years	Large RC progression resulted in worse Constant, ASES, strength, and VAS scores (*P* < 0.05)	39
Saffran, 2011 43, b	Nonsurgically treated patients with full thickness RC tears ages <60 years; secondary care hospital; 51 patients; 28 males; mean age 54 years	Patients in considerable pain at followup had an increase in tear size >5 mm (*P* = 0.002); no correlation was found between the appearance of new RC tears in the followup ultrasound and pain at the time of the followup	44
Yamaguchi, 2001 42, b	Asymptomatic RC tears in patients with symptomatic contralateral RC tears; population NR; 23 had ultrasound scans/no controls; 22 males; average age 69.8 years	Reduced function with increased pain (*P* < 0.05); no statistical significance shown between pain and function and tear progression (no statistical test undertaken)	39

a NR = not reported; SAB = subacromial bursa; RC = rotator cuff; SIS = subacromial impingement syndrome; AHD = acromiohumeral distance; WORC = Western Ontario Rotator Cuff Index; SST = Simple Shoulder Test; ASES = American Shoulder and Elbow; GH = glenohumeral; OR = odds ratio; 95% CI = 95% confidence interval; VAS = visual analog score.

b Studied imaging features as independent variables and symptoms as dependent variable.

c Studied symptoms as independent variables and imaging features as dependent variable.

**Table 5:** Longitudinal MRI scans and radiograph^a^

Author, year (ref.), test	Patient and study characteristics	Findings	Quality score
Ertan, 2015 63, MRI^b^	SIS without RC tears between March 2002 and August 2005; patients recruited from outpatients clinic; 63 patients: 3 groups of shoulder pain: no recurrence; relapsing course; chronic shoulder pain; 28 males; mean age 48 (range 28–74) years	Patients with type 1 changes on MRI (*P* = 0.038), have higher shoulder examination scores at the first evaluation and are more likely to achieve complete recovery with conservative treatment	39
Moosmayer, 2013 41, MRI^c^	Full‐thickness asymptomatic RC tears; orthopedic outpatients; 50 asymptomatic shoulders; age and sex ratio NR	Progression of muscle atrophy increased odds of symptom development, not statistically significant (OR 4.0 [95% CI 0.84–19.1]; *P* = 0.08); increased odds of symptom development with progressive RC fatty degeneration (OR 13.1 [95% CI 1.4–122]; *P* = 0.02)	67
Moosmayer, 2017 44, c	Patients reviewed by a single orthopedic surgeon in a Norwegian secondary care center; 37 patients; 30 males; average age 61 years	Worse Constant score, ASES, and muscle strength in those with supraspinatus atrophy	39
Cho, 2010 70, c	Treated calcific tendinitis; secondary care; 87 patients/no controls; 18 males; mean age 53.2 years	VAS, Constant score, UCLA scale, and ROM improved, irrespective of calcification location, deposit type, or size	28

a MRI = magnetic resonance imaging; SIS = subacromial impingement syndrome; RC = rotator cuff; NR = not reported; OR = odds ratio; 95% CI = 95% confidence interval; ASES = American Shoulder and Elbow; VAS = visual analog score; UCLA = University of California at Los Angeles; ROM = range of motion.

b Studied symptoms as independent variables and imaging features as dependent variable.

c Studied imaging features as independent variables and symptoms as dependent variable.

In 4 studies of mixed quality, an increase in RC tear was associated in the incidence of pain 39, 40, 43, 44, although this was not shown in 2 other studies 41, 42. Two high‐quality, unadjusted studies showed function worsened with increasing RC tear 39, 44. An increase in RC tear and tear type from partial to full thickness was associated with the incidence of symptoms, measured using a composite score 39, although this did not reach statistical significance in 2 other studies of mixed quality 41, 42. Overall, the high‐quality studies suggested that increasing size of tears was associated with symptom incidence 39, 40. These studies were unadjusted.

#### Tendinopathies and SAB pathology

One low‐quality, unadjusted MRI study reported that patients with tendon edema and inflammation were more likely to achieve complete recovery with conservative treatment compared to those with fibrosis or tears (*P* = 0.038) 63. One high‐quality, unadjusted ultrasound study found that pain was associated with SAB thickness 1 week after a marathon swim (*P* = 0.032) 37.

#### Calcification

One low‐quality, unadjusted ultrasound study showed that vascularity and shape were associated with resorption of the calcium deposit and improved pain (*P* < 0.001) 36. One low‐quality, unadjusted radiographic study reported no association between calcium deposition and progression of pain or function 70.

#### Other features

One high‐quality, unadjusted ultrasound study reported a reduction of the AHD narrowing on abduction correlated with improvement of symptoms 38. The rate of progression to advanced fatty muscle degeneration on MRI and the long head of the biceps on ultrasound was associated with an increased odds of symptom incidence, measured using a composite score in a high‐quality, unadjusted study 41 but not in another study 40. One study reported no relationship between the incidence of pain and progression of fatty degeneration 40. Another low‐quality, unadjusted study found supraspinatus atrophy was associated with worse strength and composite scores 44.

### Studies exploring multiple pathologies

Only 1 low‐quality, unadjusted study examined the association of combined pathologies with pain 25, and reported effusions in the SAB were associated with shoulder pain independent of the underlying pathology 25. Twenty of 56 studies evaluated more than 1 pathology, but the majority did not examine a combination of pathologies: 8 ultrasound studies (8 cross‐sectional 21, 23, 25, 32, 33, 1 longitudinal 40), 9 cross‐sectional MRI studies (15, 48, 49, 54, 55, 56, 58, 60, 62), 1 radiographic study 68, and 2 combined MRI and ultrasound studies 16, 41.

## Discussion

This systematic review is the first to comprehensively examine the relationship of imaging features with shoulder symptoms. The majority of studies reported conflicting results and evaluated single‐shoulder pathologies. RC tendons contain nociceptors, and it would be rational to expect that as tear size increased, patients would be more likely to report pain and functional impairment. However, studies evaluating RC tears reported conflicting results, and the majority of studies were unadjusted and of low quality. A cross‐sectional, high‐quality, adjusted study did not find any association with symptoms, although in high‐quality, unadjusted longitudinal studies, increasing size of tears was associated with symptom incidence. Inflammation may also be a possible cause of pain. The relationship between imaging and adhesive capsulitis was only evaluated in cross‐sectional studies. Although these studies were unadjusted, enhancement of the joint capsule on MRI and increased uptake of ^18^F‐FDG in the RC interval, acromioclavicular joint, or axillary recess on PET may be associated with symptoms. There were conflicting results on RC tendinopathy, SAB pathology, and calcific tendinopathy and symptoms. These studies were unadjusted and of mixed quality, and therefore further high‐quality studies are required to determine whether any relationship exists.

Studies have shown that numerous pathologies commonly coexist in the same symptomatic individual, although most studies in this review did not compare multiple pathologies with symptoms 75, 76. Only 1 unadjusted, low‐quality study evaluated shoulder pain with multiple pathologies in this review, and the authors found that SAB effusion may be associated with pain. Recent work suggests that these multiple pathologies may cluster into groups that could contribute to different outcomes, further confounding structure‐pain analyses 75.

In 9 of the 56 studies, the pathologies being studied were not defined, and multiple studies used varying definitions for the same pathology. These differences in nomenclature have further added to the confusion in diagnosing and treating shoulder pain 10. There was also heterogeneity between study populations, and this may be a reason for conflicting findings.

Structure‐pain relationships are complex. There is the possibility that there may be no relationship between imaging findings and symptoms, and imaging findings need to be considered as part of a wider pain construct. Other factors that may be associated with musculoskeletal symptoms include age, sex, body mass index, activity, mental health, and central sensitization 77. Only 6 studies adjusted for age and sex when evaluating the relationship between shoulder pain and imaging 15, 26, 27, 35, 51, 54, and none adjusted for psychological factors. Other adjustments included occupation 54, arm dominance 15, 27, 54, and comorbidity 15.

There were several limitations to this work. Observational studies were rated relative to the overall mean quality scores, which may have artificially rated studies as high quality. However, the distribution of quality scores indicated a broad range of quality. Publication bias could not be assessed with a funnel plot as there were insufficient results for odds and relative risk ratios. Meta‐analysis was not performed due to the heterogeneous nature of the measures of the features and pain or function outcomes.

Shoulder imaging is increasingly used for assessment of shoulder pain. Despite this rise, the relationship of imaging with symptoms and its role in informing management remain unknown. Understanding the relevance of imaging‐detected pathologies and their role in the shoulder management pathway is essential to improving care and reducing costs. This review found that increasing RC tear and certain imaging features found in adhesive capsulitis may be associated with symptoms. Further high‐quality, adjusted prospective studies evaluating the role of multiple imaging pathologies and other extrinsic factors are required to understand the role of imaging in shoulder pain care pathways.

## Author Contributions

All authors were involved in drafting the article or revising it critically for important intellectual content, and all authors approved the final version to be submitted for publication. Dr. Conaghan had full access to all of the data in the study and takes responsibility for the integrity of the data and the accuracy of the data analysis.

### Study conception and design

Tran, Cowling, Smith, Barr, Kingsbury, Conaghan.

### Acquisition of data

Tran, Cowling, Bury, Lucas, Conaghan.

### Analysis and interpretation of data

Tran, Cowling, Bury, Barr, Conaghan.
