# Supplementary material for: What Imaging‐Detected Pathologies Are Associated With Shoulder Symptoms and Their Persistence? A Systematic Literature Review
Source: Arthritis Care Res (Hoboken). 2018 Jun 6;70(8):1169–84. doi: 10.1002/acr.23554 (PMC6099421; doi:10.1002/acr.23554)
Supplement: Supplementary file 1 [file ACR-70-1169-s001.docx]

# Supplementary Table 1 Search strategy (1950 to April 2017):

| 1 | exp SCAPULA/ | 32 | exp SHOULDER IMPINGEMENT SYNDROME/ |
| --- | --- | --- | --- |
| 2 | exp SHOULDER/ | 33 | impinge*.ti,ab |
| 3 | exp HUMERUS/ | 34 | exp TENDINOPATHY/ |
| 4 | exp SHOULDER JOINT/ | 35 | tendonitis.ti,ab |
| 5 | exp ROTATOR CUFF/ | 36 | tendinitis.ti,ab |
| 6 | exp ACROMIOCLAVICULAR JOINT/ | 37 | tendonopathy.ti,ab |
| 7 | exp CLAVICLE/ | 38 | tendinopathy.ti,ab |
| 8 | scapul*.ti,ab | 39 | tenosynov*.ti,ab |
| 9 | acromio*.ti,ab | 40 | exp OSTEOARTHRITIS/ |
| 10 | gleno*.ti,ab | 41 | osteoarth*.ti,ab |
| 11 | shoulder*.ti,ab | 42 | "rotator cuff tear*".ti,ab |
| 12 | humer*.ti,ab | 43 | "calcific tend*".ti,ab |
| 13 | rotator cuff.ti,ab | 44 | exp BURSITIS/ |
| 14 | clavic*.ti,ab | 45 | burs*.ti,ab |
| 15 | subacrom*.ti,ab | 46 | frozen.ti,ab. |
| 16 | 1 OR 2 OR 3 OR 4 OR 5 OR 6 OR 7 OR 8 OR 9 OR 10 OR 11 OR 12 OR 13 OR 14 OR 15 | 47 | shoulder.ti,ab. |
| 17 | magnetic resonance.ti,ab | 48 | 46 AND 47 |
| 18 | mr*.ti,ab | 49 | "frozen shoulder".ti,ab. |
| 19 | arthrogr*.ti,ab | 50 | "adhesive capsulitis".ti,ab. |
| 20 | exp ULTRASONOGRAPHY/ | 51 | 32 OR 33 OR 34 OR 35 OR 36 OR 37 OR 38 OR 39 OR 40 OR 41 OR 42 OR 43 OR 44 OR 45 OR 48 OR 49 OR 50 |
| 21 | ultraso*.ti,ab | 52 | exp SHOULDER PAIN/ |
| 22 | exp TOMOGRAPHY/ | 53 | pain*.ti,ab |
| 23 | ct*.ti,ab | 54 | function*.ti,ab |
| 24 | tomography.ti,ab | 55 | 52 OR 53 OR 54 |
| 25 | PET*.ti,ab | 56 | 16 AND 31 AND 51 AND 55 |
| 26 | positron*.ti,ab |  |  |
| 27 | scintigraphy*.ti,ab |  |  |
| 28 | exp RADIOGRAPHY/ |  |  |
| 29 | "x-ray".ti,ab |  |  |
| 30 | radiograph*.ti,ab |  |  |
| 31 | 17 OR 18 OR 19 OR 20 21 OR 22 OR 23 OR 24 OR 25 OR 26 OR 27 OR 28 OR 29 OR 30 |  |  |

# Supplementary Table 2 – Quality scoring

| Item | Criterion | CC | CH | CS |
| --- | --- | --- | --- | --- |
| Study population | | | | |
| 1 | Recruitment from the general population | 1 | 1 | 1 |
| 2 | Selection occurred before disease onset or at a uniform point.  A uniform point was considered to be equal baseline grade of progression (e.g. Kellgren Lawrence grade) or an analysis within the same joint | 1 | 1 | 1 |
| 3 | Cases and controls drawn were from the same population | 1 |  |  |
| 4 | Participation rate >80% for cohort studies (retrospective cohort studies score zero automatically) |  | 1 |  |
| 5 | Sufficient description of baseline characteristics - must include age, gender and BMI (or height and weight) | 1 | 1 | 1 |
| 6 | Baseline characteristics comparable between cases and controls - must include age, gender and BMI (or height and weight) | 1 |  |  |
| Assessment of Imaging-detected risk factor or feature | | | | |
| 7 | Risk factor / feature assessed with a standardised method (e.g. but not a subjective opinion of a radiologist) | 1 | 1 | 1 |
| 8 | Risk factor / feature assessment was identical (performed the same way) in the studied population(s) | 1 | 1 | 1 |
| 9 | Risk factor / feature was assessed prior to the outcome (pain or function). A score of zero was allocated if the methods did not describe this. | 1 | 1 | 1 |
| Assessment of outcome (pain or function ) | | | | |
| 10 | Outcome assessment was identical in the studied population(s) | 1 | 1 | 1 |
| 11 | Outcomes were assessed reproducibly (intraclass correlation coefficient > 0.81 with a standardised assessment). If multiple outcomes were measured the mean reproducibility score was used. | 1 | 1 | 1 |
| 12 | Outcome classification was standardised (e.g. the SPADI pain score but not a subjective opinion of a patient’s pain) | 1 | 1 | 1 |
| Study design | | | | |
| 13 | Prospective study design used |  | 1 |  |
| 14 | Follow up time > 3 years | 1 | 1 |  |
| 15 | Information provided on completers vs withdrawls in cohorts (without prospective trial data cohorts automatically score zero) |  | 1 |  |
| 16 | Outcome evaluators were blinded to feature (risk factor) | 1 | 1 | 1 |
| 17 | Analysis of relationship between feature and outcome was planned prospectively | 1 | 1 | 1 |
| Analysis and data presentation | | | | |
| 18 | The frequency of most important outcomes were given | 1 | 1 | 1 |
| 19 | appropriate analysis techniques used (statistical or comparative techniques) | 1 | 1 | 1 |
| 20 | adjusted for at least age, BMI and gender | 1 | 1 | 1 |
| Maximum Score | | 17 | 18 | 14 |

CC: case control, CH cohort (prospective and retrospective), CS: cross sectional

|  |  | Quality Scoring Criteria | | | | | | | | | | | | | | | | | | | | |  |  |
| --- | --- | --- | --- | --- | --- | --- | --- | --- | --- | --- | --- | --- | --- | --- | --- | --- | --- | --- | --- | --- | --- | --- | --- | --- |
| No. | **US CROSS-SECTIONAL** | 1 | 2 | 3 | 4 | 5 | 6 | 7 | 8 | 9 | 10 | 11 | 12 | 13 | 14 | 15 | 16 | 17 | 18 | 19 | 20 | Total | | % |
|  | Ardic 2006 | 0 | 0 |  |  | 0 |  | 0 | 1 | 0 | 1 | 0 | 1 |  |  |  | 0 | 1 | 1 | 1 | 0 | 6 | | 43 |
|  | Brasseur 2004 | 0 | 0 |  |  | 0 |  | 0 | 1 | 0 | 1 | 0 | 0 |  |  |  | 0 | 1 | 1 | 1 | 0 | 5 | | 36 |
|  | Cholewinski 2008 | 0 | 0 |  |  | 0 |  | 1 | 1 | 0 | 1 | 0 | 0 |  |  |  | 0 | 1 | 1 | 1 | 0 | 6 | | 43 |
|  | Daghir 2012 | 0 | 0 |  |  | 0 |  | 0 | 1 | 0 | 1 | 0 | 0 |  |  |  | 0 | 0 | 1 | 1 | 0 | 4 | | 29 |
|  | Chiou 2002 | 0 | 0 |  |  | 0 |  | 0 | 1 | 0 | 1 | 0 | 0 |  |  |  | 0 | 0 | 1 | 1 | 0 | 4 | | 29 |
|  | Draghi 2015 | 0 | 0 |  |  | 0 |  | 0 | 1 | 0 | 1 | 0 | 0 |  |  |  | 0 | 1 | 1 | 1 | 0 | 5 | | 36 |
|  | Fehringer 2008 | 0 | 0 |  |  | 0 |  | 0 | 1 | 0 | 1 | 0 | 1 |  |  |  | 0 | 1 | 1 | 1 | 0 | 6 | | 43 |
|  | Hamid 2012 | 0 | 0 |  |  | 0 |  | 0 | 1 | 1 | 1 | 0 | 0 |  |  |  | 1 | 1 | 1 | 1 | 0 | 7 | | 50 |
|  | Joensen 2009 | 1 | 0 |  |  | 0 |  | 0 | 1 | 0 | 1 | 0 | 1 |  |  |  | 0 | 1 | 1 | 1 | 0 | 7 | | 50 |
|  | Keener 2009 | 0 | 0 |  |  | 0 |  | 0 | 1 | 0 | 1 | 1 | 0 |  |  |  | 0 | 1 | 1 | 1 | 0 | 6 | | 43 |
|  | Keener 2010 | 0 | 0 |  |  | 0 |  | 0 | 1 | 0 | 1 | 0 | 1 |  |  |  | 0 | 1 | 1 | 1 | 0 | 6 | | 43 |
|  | Le Goff 2010 | 0 | 0 |  |  | 0 |  | 0 | 1 | 1 | 1 | 0 | 0 |  |  |  | 0 | 1 | 1 | 1 | 0 | 6 | | 43 |
|  | McMahon 2014 | 0 | 0 |  |  | 0 |  | 0 | 1 | 0 | 1 | 0 | 1 |  |  |  | 1 | 1 | 1 | 1 | 0 | 7 | | 50 |
|  | Tracy 2010 | 0 | 0 |  |  | 1 |  | 0 | 1 | 0 | 0 | 1 | 0 |  |  |  | 0 | 1 | 1 | 1 | 0 | 5 | | 43 |
|  | Wu 2010 | 0 | 0 |  |  | 1 |  | 0 | 1 | 0 | 1 | 1 | 0 |  |  |  | 0 | 1 | 1 | 1 | 1 | 8 | | 57 |
|  | Yamaguchi 2006 | 0 | 0 |  |  | 0 |  | 0 | 1 | 0 | 1 | 0 | 0 |  |  |  | 0 | 1 | 1 | 1 | 0 | 5 | | 36 |

|  |  | Quality Scoring Criteria | | | | | | | | | | | | | | | | | | | |  |  |
| --- | --- | --- | --- | --- | --- | --- | --- | --- | --- | --- | --- | --- | --- | --- | --- | --- | --- | --- | --- | --- | --- | --- | --- |
| No. | **US LONGITUDINAL COHORT STUDY** | 1 | 2 | 3 | 4 | 5 | 6 | 7 | 8 | 9 | 10 | 11 | 12 | 13 | 14 | 15 | 16 | 17 | 18 | 19 | 20 | total | % |
|  | Chiou 2001 | 0 | 0 |  | 1 | 0 |  | 0 | 1 | 0 | 1 | 0 | 0 | 1 | 0 | 0 | 0 | 1 | 1 | 1 | 0 | 7 | 39 |
|  | Couanis 2015 | 0 | 1 |  | 1 | 0 |  | 0 | 1 | 1 | 1 | 0 | 0 | 1 | 0 | 1 | 0 | 1 | 1 | 1 | 0 | 10 | 56 |
|  | Desmeules 2004 | 0 | 0 |  | 1 | 0 |  | 0 | 1 | 1 | 1 | 1 | 1 | 1 | 0 | 0 | 1 | 1 | 1 | 1 | 0 | 11 | 61 |
|  | Keener 2015 | 0 | 0 |  | 1 | 0 |  | 0 | 1 | 0 | 1 | 0 | 1 | 1 | 1 | 1 | 0 | 1 | 1 | 1 | 0 | 10 | 56 |
|  | Mall 2010 | 0 | 1 |  | 1 | 0 |  | 0 | 1 | 1 | 1 | 1 | 1 | 1 | 0 | 0 | 0 | 1 | 1 | 1 | 0 | 11 | 61 |
|  | Saffran 2011 | 0 | 0 |  | 0 | 0 |  | 0 | 1 | 1 | 1 | 0 | 0 | 1 | 0 | 1 | 1 | 0 | 1 | 1 | 0 | 8 | 44 |
|  | Yamaguchi 2001 | 0 | 0 |  | 0 | 0 |  | 0 | 1 | 1 | 1 | 0 | 1 | 0 | 0 | 0 | 0 | 1 | 1 | 1 | 0 | 7 | 39 |

|  | | Quality Scoring Criteria | | | | | | | | | | | | | | | | | | | |  |  |
| --- | --- | --- | --- | --- | --- | --- | --- | --- | --- | --- | --- | --- | --- | --- | --- | --- | --- | --- | --- | --- | --- | --- | --- |
| No. | **PET CROSS-SECTIONAL** | 1 | 2 | 3 | 4 | 5 | 6 | 7 | 8 | 9 | 10 | 11 | 12 | 13 | 14 | 15 | 16 | 17 | 18 | 19 | 20 | total | % |
|  | Kim 2013 | 0 | 0 |  |  | 0 |  | 1 | 1 | 0 | 1 | 0 | 0 |  |  |  | 0 | 1 | 1 | 1 | 0 | 6 | 43 |
|  | Sridhiran 2017 | 0 | 0 |  |  | 0 |  | 0 | 0 | 0 | 0 | 0 | 0 |  |  |  | 1 | 0 | 0 | 1 | 0 | 2 | 14 |

|  | | Quality Scoring Criteria | | | | | | | | | | | | | | | | | | | |  |  |
| --- | --- | --- | --- | --- | --- | --- | --- | --- | --- | --- | --- | --- | --- | --- | --- | --- | --- | --- | --- | --- | --- | --- | --- |
| No. | **BONE SCAN CROSS SECTIONAL** | 1 | 2 | 3 | 4 | 5 | 6 | 7 | 8 | 9 | 10 | 11 | 12 | 13 | 14 | 15 | 16 | 17 | 18 | 19 | 20 | total | % |
|  | Koike 2013 | 0 | 0 |  |  | 0 |  | 1 | 1 | 0 | 1 | 1 | 1 |  |  |  | 1 | 1 | 1 | 1 | 0 | 7 | 50 |
|  | Clunie 1998 | 0 | 0 |  |  | 0 |  | 0 | 1 | 0 | 1 | 0 | 0 |  |  |  | 0 | 0 | 0 | 0 | 0 | 2 | 14 |
|  | Binder 1984 | 0 | 0 |  |  | 0 |  | 1 | 0 | 1 | 0 | 0 | 0 |  |  |  | 0 | 0 | 1 | 0 | 0 | 3 | 21 |

|  |  | Quality Scoring Criteria | | | | | | | | | | | | | | | | | | | |  |  |
| --- | --- | --- | --- | --- | --- | --- | --- | --- | --- | --- | --- | --- | --- | --- | --- | --- | --- | --- | --- | --- | --- | --- | --- |
| No. | **X-RAY CASE-CONTROL STUDY** | 1 | 2 | 3 | 4 | 5 | 6 | 7 | 8 | 9 | 10 | 11 | 12 | 13 | 14 | 15 | 16 | 17 | 18 | 19 | 20 | total | % |
|  | Endo 2001 | 0 | 0 | 1 |  | 0 | 0 | 1 | 1 | 0 | 1 | 1 | 1 |  | 0 |  | 0 | 0 | 1 | 1 | 0 | 8 | 47 |

|  |  | Quality Scoring Criteria | | | | | | | | | | | | | | | | | | | |  |  |
| --- | --- | --- | --- | --- | --- | --- | --- | --- | --- | --- | --- | --- | --- | --- | --- | --- | --- | --- | --- | --- | --- | --- | --- |
| No. | **X-RAY CROSS-SECTIONAL** | 1 | 2 | 3 | 4 | 5 | 6 | 7 | 8 | 9 | 10 | 11 | 12 | 13 | 14 | 15 | 16 | 17 | 18 | 19 | 20 | total | % |
|  | Kircher 2010 | 0 | 0 |  |  | 0 |  | 0 | 0 | 1 | 1 | 1 | 1 |  |  |  | 0 | 1 | 0 | 1 | 0 | 6 | 43 |
|  | Kircher 2012 | 0 | 0 |  |  | 0 |  | 0 | 1 | 0 | 1 | 1 | 1 |  |  |  | 0 | 1 | 1 | 1 | 0 | 7 | 50 |
|  | Mayerhoefer 2009 | 0 | 0 |  |  | 0 |  | 0 | 1 | 1 | 1 | 1 | 1 |  |  |  | 0 | 1 | 1 | 1 | 0 | 8 | 57 |
|  | Yamaguchi 2000 | 0 | 0 |  |  | 0 |  | 1 | 1 | 0 | 0 | 1 | 0 |  |  |  | 0 | 1 | 0 | 1 | 0 | 5 | 36 |

|  |  | Quality Scoring Criteria | | | | | | | | | | | | | | | | | | | |  |  |
| --- | --- | --- | --- | --- | --- | --- | --- | --- | --- | --- | --- | --- | --- | --- | --- | --- | --- | --- | --- | --- | --- | --- | --- |
| No. | **X-RAYS COHORT STUDY** | 1 | 2 | 3 | 4 | 5 | 6 | 7 | 8 | 9 | 10 | 11 | 12 | 13 | 14 | 15 | 16 | 17 | 18 | 19 | 20 | total | % |
|  | Cho 2010 | **0** | **0** |  | **0** | **0** |  | **0** | **0** | **0** | **1** | **0** | **1** | **0** | **0** | **0** | **0** | **1** | **1** | **1** | **0** | **5** | **28** |

|  |  | Quality Scoring Criteria | | | | | | | | | | | | | | | | | | | | |  |  |
| --- | --- | --- | --- | --- | --- | --- | --- | --- | --- | --- | --- | --- | --- | --- | --- | --- | --- | --- | --- | --- | --- | --- | --- | --- |
| No. | **MRI CROSS-SECTIONAL STUDY** | 1 | 2 | 3 | 4 | 5 | 6 | 7 | 8 | 9 | 10 | 11 | 12 | 13 | 14 | 15 | 16 | 17 | 18 | 19 | 20 | Total | | % |
|  | Ahn KY 2012 | 0 | 0 |  |  | 0 |  | 0 | 1 | 1 | 1 | 1 | 0 |  |  |  | 0 | 1 | 1 | 1 | 0 | 7 | | 50 |
|  | Birtane 2001 | 0 | 0 |  |  | 0 |  | 0 | 0 | 0 | 1 | 0 | 1 |  |  |  | 0 | 1 | 1 | 1 | 0 | 5 | | 36 |
|  | Curry EJ 2015 | 0 | 0 |  |  | 1 |  | 1 | 1 | 0 | 1 | 0 | 1 |  |  |  | 1 | 1 | 1 | 1 | 1 | 10 | | 71 |
|  | Di Mario 2005 | 0 | 0 |  |  | 0 |  | 1 | 1 | 1 | 1 | 0 | 0 |  |  |  | 0 | 0 | 1 | 0 | 0 | 5 | | 36 |
|  | Epstein R 1993 | 0 | 0 |  |  | 0 |  | 1 | 1 | 0 | 0 | 0 | 0 |  |  |  | 0 | 1 | 1 | 1 | 0 | 5 | | 36 |
|  | Gill 2014 | 1 | 0 |  |  | 0 |  | 0 | 1 | 0 | 1 | 0 | 1 |  |  |  | 0 | 0 | 1 | 1 | 0 | 6 | | 43 |
|  | Hodgson RJ 2012 | 0 | 0 |  |  | 0 |  | 1 | 1 | 0 | 1 | 1 | 1 |  |  |  | 0 | 1 | 1 | 1 | 0 | 8 | | 57 |
|  | Jung 2013 | 0 | 0 |  |  | 0 |  | 0 | 0 | 0 | 0 | 0 | 0 |  |  |  | 0 | 0 | 1 | 0 | 0 | 1 | | 7 |
|  | Kanatli U 2013 | 0 | 0 |  |  | 0 |  | 1 | 1 | 1 | 1 | 1 | 1 |  |  |  | 1 | 1 | 1 | 1 | 0 | 10 | | 71 |
|  | Krief OP 2006 | 0 | 0 |  |  | 0 |  | 1 | 1 | 0 | 1 | 0 | 1 |  |  |  | 0 | 1 | 1 | 1 | 0 | 7 | | 50 |
|  | Moses DA 2006 | 0 | 0 |  |  | 0 |  | 1 | 1 | 0 | 1 | 0 | 0 |  |  |  | 0 | 1 | 1 | 1 | 0 | 6 | | 42 |
|  | Reuter 2008 | 0 | 0 |  |  | 0 |  | 0 | 1 | 0 | 1 | 0 | 0 |  |  |  | 0 | 1 | 1 | 1 | 0 | 5 | | 36 |
|  | Song 2011 | 0 | 0 |  |  | 0 |  | 0 | 1 | 0 | 1 | 1 | 0 |  |  |  | 1 | 1 | 1 | 1 | 0 | 7 | | 50 |
|  | Unruh 2014 | 0 | 0 |  |  | 0 |  | 0 | 0 | 1 | 1 | 0 | 1 |  |  |  | 0 | 1 | 1 | 1 | 0 | 6 | | 43 |
|  | White 2006 | 0 | 0 |  |  | 0 |  | 0 | 0 | 0 | 0 | 0 | 0 |  |  |  | 0 | 1 | 1 | 1 | 0 | 3 | | 21 |
|  | Williamson 1994 | 0 | 0 |  |  | 0 |  | 0 | 1 | 0 | 0 | 0 | 0 |  |  |  | 0 | 0 | 0 | 1 | 0 | 2 | | 14 |

|  |  | Quality Scoring Criteria | | | | | | | | | | | | | | | | | | | |  |  |
| --- | --- | --- | --- | --- | --- | --- | --- | --- | --- | --- | --- | --- | --- | --- | --- | --- | --- | --- | --- | --- | --- | --- | --- |
| No. | **MRI COHORT STUDY** | 1 | 2 | 3 | 4 | 5 | 6 | 7 | 8 | 9 | 10 | 11 | 12 | 13 | 14 | 15 | 16 | 17 | 18 | 19 | 20 | total | % |
|  | Moosmayer S 2013 | 0 | 1 |  | 1 | 0 |  | 1 | 1 | 0 | 1 | 0 | 1 | 1 | 1 | 0 | 1 | 1 | 1 | 1 | 0 | 12 | 67 |
|  | Moosmayer 2017 | 0 | 0 |  | 0 | 0 |  | 0 | 1 | 0 | 0 | 0 | 1 | 0 | 1 | 1 | 1 | 0 | 1 | 1 | 0 | 7 | 39 |
|  | Ertan 2015 | 0 | 0 |  | 0 | 1 |  | 0 | 0 | 0 | 1 | 0 | 1 | 0 | 1 | 0 | 0 | 1 | 1 | 1 | 0 | 7 | 39 |

| No. | **MRI CASE CONTROL STUDY** | 1 | 2 | 3 | 4 | 5 | 6 | 7 | 8 | 9 | 10 | 11 | 12 | 13 | 14 | 15 | 16 | 17 | 18 | 19 | 20 | total | % |
| --- | --- | --- | --- | --- | --- | --- | --- | --- | --- | --- | --- | --- | --- | --- | --- | --- | --- | --- | --- | --- | --- | --- | --- |
|  | Frost P 1999 | 0 | 1 | 1 |  | 0 | 0 | 1 | 1 | 0 | 1 | 0 | 1 |  | 0 |  | 1 | 1 | 1 | 1 | 0 | 10 | 59 |
|  | Graichen H 1999 | 0 | 0 | 0 |  | 0 | 0 | 1 | 1 | 1 | 1 | 0 | 1 |  | 0 |  | 0 | 1 | 1 | 1 | 0 | 8 | 47 |
|  | Schweitzer M 1995 | 0 | 0 | 1 |  | 0 | 0 | 0 | 1 | 1 | 1 | 0 | 0 |  | 0 |  | 1 | 1 | 1 | 1 | 0 | 8 | 47 |

# Supplementary Table 3 – Definition of pathology

| Pathology | Definitions |
| --- | --- |
| MRI defined partial rotator cuff tear | Incomplete tears are either intratendinous or partial. In intratendinous tears, the split is only within the tendon itself. In partial tears, some tendinous fibers on the articular or bursal surface are interrupted (16)  Tear size was categorized into partial-thickness tear, <2 cm full-thickness tear, and ≥2 cm full-thickness tear (45)  A partial tear was defined as T2 signal of defect was a fluid signal (49)  “bridging sign” , a band-like structure connecting cranial portion of the subscapularis tendon and anterior margin of the supraspinatus tendon through the subcoracoid and subacromial space with intermediate to low signal intensity in all sequences, which is associated with subscapularis tendon tear (52)  Classification according to which surface of the tendon is involved and grades the severity of the tear according to its depth: grade 0, homogeneous signal and regular margins of tendon; grade 1, articular or bursal surface side lesion involving less than a quarter of the tendon thickness; grade 2, partial-thickness tear involving less than half the thickness of the tendon; and grade 3, partial-thickness tear involving more than one half of the thickness of the tendon with tenuous continuity but without full-thickness tear (54)  The main criteria for partial-thickness tears were focal heterogenous hypoechogenicity and incomplete hypoechoic clefts. Secondary signs – including double cortex sign, pitting and irregularity of the bony surface of the greater tuberosity, and fluid in the biceps tendon sheath and the subdeltoid bursa – were used as diagnostic aids but were regarded as insufficient to make the diagnosis alone (41)  A partial tear was a partial thickness defect on the coronal and/or sagittal images (58)  The supraspinatus tendon was considered torn when there was increased signal within the tendon on both proton density and T2 weighted images, or when there was disruption and retraction of the tendon seen on any pulse sequence (62) |
| Ultrasound defined partial rotator cuff tear | Partial tear of supraspinatus tendon was defined as the presence of an anechoic or hypoechoic area, containing or not a central hyper- echoic core, only partly involving the thickness of the tendon at either the joint or above it (21)  Partial rupture of subscapularis or infraspinatus tendon was defined as part of the tendon still attached to its insertion; thus, the echostructure of the rotator-cuff muscles was assessed (21)  Rotator cuff integrity was evaluated according to the modified 5-grade Wiener and Seitz classification:  Type III - area of cuff discontinuity at the inner or outer side of the cuff tendons; local loss of ‘‘anterior arc’’ of the cuff shape or major hypo-echoic area within the cuff. This type corresponds to partial full-thickness tear (23)  A partial-thickness tear was recorded when there was minimal flattening of the bursal side of the rotator cuff or when a distinct hypoechoic or mixed hypoechoic defect was visualized in both the longitudinal and transverse planes (35)  Tears measuring ≤15 mm were considered to only involve supraspinatus (or 1 tendon), tears that measured >15 mm but ≤30 mm were considered to be involving supraspinatus and infraspinatus and tears that measured >30mm in the transverse dimension were considered to involve supraspinatus, infraspinatus and part of teres minor (35) |
| MRI defined complete rotator cuff tear | A complete rotator cuff tear was defined as a focal, well-defined area of increased signal intensity on T1-weighted and T2-weighted images that ex- tended through the entire thickness of tendon. Complete tears are either focal (scoring as 1), subtotal (scoring as 2), or total (scoring as 3). Focal tears display a piercing tendon hole; in subtotal tears, only a few fibers are regularly inserted, whereas in total tears, all tendon fibers are torn and the stump is retracted under the acromion (16)  Tear was categorized as full-thickness if tear ≥2 cm (45)  Criteria adapted from Zlatkin et al: Grade 0 = normal; grade 1 = tendinitis; grade 2 = degeneration; grade 3 full thickness tear (48)  Full-thickness tear, there was a tear evident from one side to other side of the tendon but not necessarily whole tendon, for a complete tear all fibres of the tendon were torn (49)  Criteria for full-thickness tears included nonvisualisation of the rotator cuff, hypoechoic or anechoic discontinuity, and contour concavity of the superiorborder of the rotator cuff tendon (41)  Measurement of tear size was made along a measuring line drawn between the edges of the tear (anterior-posterior plane) or between the lateral margin of the tear and the greater tuberosity (medial-lateral plane). Tears were classified into 3 groups according to tear size progression in the anterior-posterior plane: no to small progression (−5 to +9.9 mm), medium progression (10 to 19.9 mm), and large progression (≥20 mm) (44)  A full thickness tear was categorized as a complete interruption of the tendon on the coronal and/or sagittal images (58)  The diagnosis of a full-thickness rotator cuff tear was made when a high-signal-intensity, well-defined abnormality was seen in the rotator cuff with or without subacromial bursal fluid or retraction (59) |
| Ultrasound defined complete rotator cuff tear | The criteria of van Holsbeck and Introcaso for full thickness rotator cuff tears was used (with added scores): a discontinuity in the rotator cuff (scored as 1) and extension from the bursal to the humeral side of the rotator cuff (scored as 2) (16)  A complete tear or rupture of the supraspinatus tendon was defined by one of the following criteria: (a) anechoic area through the entire thickness of the tendon; (b) a flat part of the superficial contour of the tendon; (c) a rotator cuff <2 mm thick or (d) no cuff visible (21)  The diagnostic criterion for a complete rupture of the subscapularis or infraspinatus tendon was no visibility of the tendon at its insertion site (21)  Rotator cuff integrity was evaluated according to the modified 5-grade Wiener and Seitz classification: Type IV - hypoechoic linear zone extending through the entire thickness of the cuff; segmental loss of convex cuff contour; the deltoid muscle may be found pushed into the cuff defect—to the degree where it is in contact with the humeral head; visualisation of the hyaline cartilage under- lying the cuff tendons ‘‘naked cartilage sign”. Type V: non-visualization of the rotator cuff tendons. Subdeltoid fascia and the deltoid muscle apposed to the contour of humeral head (23)  The criteria of a rotator cuff tear included the following: (1) nonvisualization of the rotator cuff; (2) a focal hypoechoic cleft in the rotator cuff; (3) focal thinning of the rotator cuff; (4) focal depression of the rotator cuff; and (5) a focal heterogeneous, hypoechoic rotator cuff with a subdeltoid bursa (22)  Full- thickness tears were classified by size (based on tear width or length, whichever was larger) as small (<10 mm), medium (10 to 30 mm) or large (>30 mm) (29)  A full-thickness rotator cuff tear was recorded when the rotator cuff could not be visualized because of complete avulsion and retraction under the acromion or when a focal defect in the rotator cuff was created by a variable degree of retraction of the torn tendon edges (Safran 2011).  A full-thickness rotator cuff tear was recorded when the rotator cuff could not be visualized because of complete avulsion and retraction un- der the acromion or when a focal defect in the rotator cuff was created by a variable degree of retraction of the torn tendon edges (35) |
| Arthrography defined rotator cuff tear | Rupture of the rotator cuff was considered to be present in an immediate flow of contrast medium from the shoulder joint into the subacromial/subdeltoid bursa occurred. With contrast medium present in the shoulder joint and subacromial bursa, the rotator cuff was clearly visible above the humeral head (65) |
| Capsulitis | Capsulitis was defined on arthrography as a marked reduction in joint volume (often under 5 ml) with loss of distensibility of the shoulder joint was found. Marked irregularity of joint outline and early lymphatic filling was also sometimes seen (65)  Unilateral adhesive capsulitis is defined as ≥50 % loss of movement of the shoulder joint relative to the non-affected side in one or more of three movement directions (i.e., forward elevation, external rotation in 0° of abduction, or internal rotation). To determine joint capsule thickness, combined capsular and synovial thicknesses of the axillary pouch were obtained by measuring the widest portion of the capsule, which was determined based on the distance between the high signal fluid in the axillary recess and the outer border of the capsule in perpendicular direction to the capsular configuration on T2-weighted fat suppressed oblique coronal images. Gadolinium enhancement of the joint capsule in the axillary recess was assessed on T1-weighted fat-suppressed oblique coronal images and graded by the intensity and extent of the enhancement as follows: mild, subtle enhancement of the capsule with insufficient intensity; moderate, sufficiently strong enhancement of the capsule involving less than half of the capsule circumference; severe, sufficient enhancement involving over the half of the capsule circumference (15) |
| MRI defined Biceps Tendon Pathology | Biceps tendon lesions were classified as grade zero when the tendon had a normal shape and signal in all planes, grade 1 when shape abnormalities or signal abnormalities without discontinuity were present, and grade 2 when a discontinuity of the tendon was observed (54)  Tendinopathy was categorized by abnormal signal intensity on short TE images without a defect in the tendon in fluid sensitive images (58)  Normal fluid in the biceps tendon sheath was 1-2mm in thickness (59) |
| Ultrasound defined biceps tendon pathology | The location of the LHB tendon with respect to the intertubercular groove was considered normal when lying within the groove, in subluxation when it was situated beyond the medial margin of the groove, and in luxation when it was outside the groove (21) |
| MRI defined Tendon Pathology | Tendonosis was present if the proton density fat-saturated sequence signal was increased but the T2 signal was less than that obtained if fluid was present (49)  Rotator cuff tear based on the number of tendons  involved; retraction of the rotator cuff tear in the coronal plane (minimal retraction, mid-humeral retraction, glenohumeral retraction, or retraction to glenoid); and the degree of muscle atrophy. (56)  In calcific tendinitis, classification and measurement of size of the calcium deposit and the measurements for the calculation of the acromion index were made at standardized true antero-posterior radiographs using digital X-rays with resolution of 0.1 mm. The deposits were classified according to Gärtner (appearance) and Bosworth (size) (68)  For rotator cuff pathologies, especially the supraspinatus tendon, MRI findings were graded as normal, tendinosis, or a partial-thickness tear of the supraspinatus tendon (15)  Zlatkin’s MRI stages of SIS = stage 0 = normal tendon morphology and signal intensity; stage 1 = increased signal intensity without thinning irregularity or discontinuity; stage 2 = increased signal intensity with thinning irregularity; stage 3 = complete disruption of supraspinatus tendon (64)  Tendon retraction in the coronal plane was classified in stages as described by Boileau et al. (45)  The MRI classification was a modified version of the MRI staging system of Zlatkin et al: Type 1 showed increased signal intensity due to edema and inflammation of the tendon. Type 2 showed findings suggestive of fibrosis and tendinitis on MRI, in addition to the findings described for type 1. Type 2 was further subdivided into type 2a, in which there was involvement of the articular surface of the tendon, and type 2b, in which there was involvement of the bursal surface. Type 3 showed a partial tear of the rotator cuff tendon. Finally, type 4 showed a complete tear of the tendon (63)  The rotator interval was assessed with respect to signal abnormality in the subcoracoid fat, which was considered to be present if there was a discrete focus of homogeneous low signal within the rotator interval on an oblique sagittal T1- weighted image. Subcoracoid fat obliteration was graded subjectively as absent, partial, or complete (15) |
| Ultrasound defined tendon pathology | Calcifications were identified as hyperechoic linear, round, or oval areas interrupting the US wave (21)  The categorization of the phase of calcification as formative or resorptive was determined by the presence of symptoms; patients with acute onset of moderate or severe pain were classified as having calcification in the resorptive phase; otherwise, the calcification was classified as being in the formative phase (22)  Tendon thickness was measured by the built-in calibrated callipers at predefined sites using osseous land- marks for exact positioning of the transducer longitudinally to the tendon. Thickness was measured on an imaginary line placed at 90◦ to the skin surface and drawn to the superficial and deep margins of the paratenon. The exact anatomical sites were determined by the deep anchor of the imaginary line; the base of the tuberculum majus plateau of the os humeri (28)  Tear width describes the anterior-to-posterior dimension and tear length, the medial- to-lateral dimension. The tear area was calculated by multi- plying the tear width by the tear length. (30)  Calcification of the rotator cuff was diagnosed if a hyperechogenic focus with or without acoustic shadow was found within the supraspinatus, infraspinatus or subscapularis tendon on ultrasound (31) |
| Radiography/bone isotope defined tendon pathology | The size of a calcific deposit was defined by multiplying the long axis by the short axis. The calcific deposits were classified into type I (sharply outlined and densely structured), type II (sharply outlined and inhomogeneous or homogenous with no defined border), and type III (cloudy and transparent in structure) (70)  A diagnosis of a rotator cuff tear when at least 3 of these 4 criteria were met: (1) flattening of peribursal fat, (2) loss of actual rotator cuff tendon, (3) surface irregularity of the greater tuberosity, and (4) effusion of the subacromial bursa (72) |
| MRI defined Bursa Pathology | Mild subacromial bursitis had a sliver of fluid present or a small increase in T2 signal; moderate bursitis, clear fluid or thickening present; and severe bursitis, marked fluid distension and synovial thickening and/or the presence of rice bodies (49)  Images were scored on a scale of 0-2 depending on the maximum thickness of enhancing tissue: 0, no abnormal enhancement; 1, up to 3mm thickness of enhancing tissue; 2, 3mm or more enhancing tissue (51)  The criteria for subacromial bursitis was the presence of a fluid collection and enhancement in the subacromial bursa on oblique coronal T2-weighted images and oblique coronal fat-suppressed enhanced T1-weighted images (60) |
| Ultrasound defined bursa pathology | An effusion of subacromial–subdeltoid bursa (SSB) was defined as an anechoic lamina between the folds of the bursa. It was considered to be mild (lamina thickness <3 mm) or abundant (lamina thickness >3 mm) (21)  Subacromial–subdeltoid bursa thickness was considered to be abnormal when the hypoechoic lamina located between the deep hyperechoic lamina of the deltoid and the superficial hyper- echoic lamina of the supraspinatus was >2 mm thick. It was considered to be mildly thickened when this lamina was 2 or 3 mm, and markedly thickened when >3 mm (21)  Effusion in the SASD bursa was evaluated by using a binary system (yes vs. no) (25)  The patient was considered to present bursal effusion or bursitis if the width of the bursa exceeded 2mm (31) |
| Joint Effusion | A joint effusion was defined as an anechoic lamina partly, or completely surrounding the long head of the biceps tendon at the level of the intertubercular groove (21)  A glenohumoral effusion was defined as a hypoechogenic area between the posterior labrum and the infraspinatus tendon (31)  The glenohumeral joint effusion was scored on oblique coronal T2-weighted images using a modified MRI classification scheme developed by Schweitzer et al. as follows: 0, no joint fluid; 1, mild loculated fluid signal; or 2, extensive fluid signal (60)  Glenohumoral Joint fluid was graded as follows:  0 =a thin intraarticular rim without distension of the recesses  1 = either slight distension of the subscapularis recess, fluid in the biceps tendon sheath (>2mm thickness), or fluid in the axillary recess, which causes a U shape and is seen in more than one coronal oblique image  2 = distension of two of these recesses  3 = increased fluid I all three of these synovial structures (59) |
| Acromioclavicular Joint Pathology | ACJ arthritis severity was determined according to the degree of osteophytes, joint effusion, synovial thickening, bone edema and articular cartilage thinning (49)  The criteria for osteoarthritis of the acromioclavicular joint was the presence of a fluid collection and enhancement in the acromioclavicular joint on oblique coronal T2-weighted images and oblique coronal fat-suppressed enhanced T1-weighted images (60) |
| Acromion Pathology | Bigliani et al. classified the morphology of the acromion according to the acromial under surface: flat, curved, hooked and convex. The intrinsic acromion angle was measured at the intersection of two lines: one joining the anterior and posterior margins of the acromial under surface, and the other running parallel to the longitudinal axis of the acromion. The acromio-humeral distance was measured in millimetres between the most caudal point of the acromial undersurface and the most cranial point of the proximal humeral epiphysis in the acromio-clavicular joint (46)  Acromion was classified as flat (type 1), smoothly curved (type 2), or hooked (type 3). The acromions were classified according to their appearance on the T2 weighted MRI image obtained just lateral to the acromioclavicular joint (47)  All shoulders were classified into one of three acromial shapes using the SOV. The Bigliani classification system was used. This system classifies the shape of the undersurface of the acromion as flat (type I), curved (type II), or hooked (type III).  The SOV was also used to assess for the presence of an acromial spur. A spur was defined as a boney projection along the insertion of the coracoacromial ligament that showed an abrupt change in the curvature of the anterior edge of the acromion as described by Ogawa et al. The acromial index (AI) is a method to quantify the amount of lateral extension of the acromion relative to the humeral head. Described by Nyffeler et al, this calculation is done using the true anteroposterior radiograph (27)  The acromion index (AI) was calculated as described by Nyffeler et al. by dividing the distance from the glenoid plane to the most lateral aspect of the acromion (GA) by the distance from the glenoid plane to the most lateral aspect of the proximal humeral head (GH) in the true anteroposterior view (68)  Acromial shape was assessed on outlet view radiographs based on the illustrations by Toivonen et al and the criteria of Epstein et al. A flat acromion with no signs of anterior downsloping was classified as type I; an acromion with anterior downsloping in its midsection was classified as type II, and an acromion with downsloping in its anterior third as type III. If there was a bony spur on the anterior or anterolateral undersurface, the acromion was also graded as type III.  AHD is considered pathological if the minimal distance between the dense cortical bone at the inferior aspect of the acromion and the subchondral lamina of the humeral head is ≤7 mm (55) |
| Rotator Cuff Tear progression/enlargement | A full-thickness cuff tear was considered to have enlarged if its size had increased by 5 mm in any dimension compared with baseline (39)  A partial-thickness tear was considered to have enlarged when it had converted to a full-thickness defect, defined as a complete disruption of tendon continuity at the insertion (39)  Substantial tear progression was defined as transformation of a partial-thickness tear into a full-thickness tear or a size increase of >5 mm in either the width or the length of a full- thickness tear compared with that at the time of enrollment (40)  Increase in size in rotator cuff tear was defined as greater than 5mm on either longitudinal or transverse ultrasound views (42) |
| Labarum/ligament Pathology | Gleno-humoral joint cartilage damage was classified as mild if there were small areas of cartilage thinning (<50 mm thickness), moderate if areas of cartilage thinning were >50 mm or more extensive involvement of <50 mm areas of thinning and severe if there were larger areas of full cartilage loss. Glenoid labrum tears were deemed to be small if they were less than full thickness of the labrum and not displaced; large tears were full thickness (49).  Abnormal gleno-humoral Joint radiography is defined as evidence of joint space loss, osteophytosis, or notches. (73).  Maximal superior coracoacromial ligament (CAL) displacement was recorded from a measured line connecting the acromion and the coracoid process at the CAL attachment. (34). |
| Muscle Atrophy | Atrophy of the supraspinatus muscle was assessed by the tangent sign as described by Zanetti et al (41)  Atrophy of the supraspinatus muscle was assessed by the tangent sign as described by Zanetti et al. Fatty degeneration of the supraspinatus, infraspinatus, and subscapularis muscles was classified according to Goutallier et al. and Fuchs et al. The original five grades were dichotomized. Muscles showing no fat (grade 0) or only some fatty streaks (grade 1) were compared with those showing more than some fatty streaks but still more muscle than fat (grade 2), equal amounts of muscle and fat (grade 3), or less muscle than fat (grade 4) (41)  To evaluate fatty degeneration of the rotator cuff muscles, the echogenicity and echotexture of each cuff muscle was examined with use of a 3-point scale as described by Strobel et al. The echogenicity was graded in comparison with the echogenicity of the overlying muscle (i.e., the deltoid for supraspinatus grading and the trapezius for infraspinatus grading). The echotexture was graded on the basis of the visibility of the central tendon and the normal muscular pennate pattern. The sum of the echogenicity and echotexture grades was calculated and used for data analysis (40)  Fatty infiltration was determined based on the classification described by Goutallier et al, and classification of muscle atrophy was based on that by Warner et al. (45) |
| Calcific Plaque Morphology | The morphology of calcific plaque of the shoulder on HRUS was classified into 4 types, including arc-shape (echogenic arc with clear shadowing); fragmented (at least two separated echogenic plaques with or without shadowing) or punctuated (tiny calcific spots without shadowing); nodular (echogenic nodule without shadowing); and cystic types (bold echogenic wall with echo-free content) (22, 36)  Calcific shoulder plaques were classified into four types on the basis of morphology, as previously described by Chiou et al 2001 (31) |
| Subacromial Space | The ultrasonographic measurement of the subacromial space was defined as the tangential distance between the humeral head and the edge of the acromion visible on the longitudinal sonogram as hyperechoic bony landmarks when the image was frozen. The measurement obtained therefore represents the AHD at the inlet of the sub- acromial space (38)  For quantification of the subacromial space width, the minimal spatial acromiohumeral and claviculohumeral distances were determined by 3D Euclidean distance transformation (50)  Abnormal acromio-humoral space defined as <7mm (73) |
| Osteoarthritis | The degree of osteoarthritis was graded according to Samilson and Prieto by the size of the caudal osteophyte (grade I: <3 mm; II: 3–7 mm; III: >7 mm) (67)  The presence of GHJ OA was determined using the Samilson-Prieto classification (49)  For evidence of osteoarthritis, the presence of osteophytes was used, which were only considered present if they contained marrow (59) |
| Factors linked with impingement | The point of inter-section of the upper border of the scapular spine and the acromio-clavicular joint was defined as the outside point, and the medial end of the upper border of the scapular spine the inside point.  The scapular upward rotation angle (SURA), that is, the angle of tilt between the scapular spine line and the horizontal, was adopted as the parameter of the upward rotational tilt.  In order to evaluate the axial rotation tilt of the scapula, the distance between the scapular spine line and the upper border of the coracoid process was defined as the coracoid upward shift distance (CUSD) (66)  The acromio-glenoid angle (AGA) was measured as described by Banas et al. This is the angle between the inferior outline of the acromion and the superior and inferior margins of the glenoid lab- rum. Supraspinatus-glenoid angle (SGA) was measured as described by Tetreault et al. on an oblique coronal image just posterior to the acromio-clavicular joint. This is the angle formed by the supraspinatus fossa and the labral outline of the glenoid on the oblique coronal image (53)  Acromial index (AI) was measured as described by Nyff- eler et al. After obtaining a true A-P X-ray view, the measurement technique requires drawing three parallel lines and measuring the distances between those lines. The first line connects the superior and inferior osseous margins of the glenoid cavity and represents the plane of the glenoid surface. The second line is drawn tangential to the lateral border of the acromion, and the third line is drawn tangential to the most lateral part of the proximal humerus. The distance from the glenoid to the acromion is divided by the distance from the glenoid to the lateral aspect of the humeral head, and the resultant value is called the acromial index (53)  Since the coracoacromial ligament (CAL) degeneration has been proposed as a well-known indicator of the subacromial impingement, it was used to assess the subacromial impingement. The degree of CAL degeneration was assessed arthroscopically according to Royal Berkshire Hospital Classification (RBHC) as described by Levy et al. This classification system categorizes the pathology into four different grades. Normal appearance of the CAL is accounted as grade 0, minor fraying as grade I, major fraying as grade II, and visualization of the bare bone under the CAL is accounted as grade III (53)  The coracohumoral interval was defined as the interval between the coracoid process and the lesser tuberosity of the humerus was measured 3 separate times (33) |
| Glenohumoral Motion | The geometric center of the humeral head was found with the use of the center point of a “best- fit” circle positioned on the humeral articular surface. The superior and inferior end points of the glenoid articular surface were then marked to demarcate the glenoid line and the center point automatically determined by the software. A line drawn along the long axis of the humerus was compared with the glenoid line to calculate the glenohumeral angle. The perpendicular distance from the center of the humeral head to a perpendicular line drawn from the center of the glenoid line was calculated by the software for each arm abduction angle. The arm abduction angle was compared with the measured glenohumeral angle (69) |
